# Anticancer Activity of Roburic Acid: In Vitro and In Silico Investigation

**DOI:** 10.3390/ijms26136420

**Published:** 2025-07-03

**Authors:** Adrianna Gielecińska, Mateusz Kciuk, Somdutt Mujwar, Johannes A. Schmid, Renata Kontek

**Affiliations:** 1 Department of Molecular Biotechnology and Genetics, University of Lodz, Banacha 12/16, 90-237 Lodz, Poland; adrianna.gielecinska@edu.uni.lodz.pl (A.G.); mateusz.kciuk@biol.uni.lodz.pl (M.K.); 2 Doctoral School of Exact and Natural Sciences, University of Lodz, Banacha Street 12/16, 90-237 Lodz, Poland; 3 Chitkara College of Pharmacy, Chitkara University, Rajpura 140401, Punjab, India; somduttmujwar@gmail.com; 4 Institute of Vascular Biology and Thrombosis Research, Center for Physiology and Pharmacology, Medical University of Vienna, Schwarzspanierstrasse 17, 1090 Vienna, Austria; johannes.schmid@meduniwien.ac.at

**Keywords:** roburic acid, colorectal cancer, antiproliferative activity, inflammation, NF-κB signaling

## Abstract

Natural compounds remain a valuable source of anticancer agents due to their structural diversity and multi-targeted mechanisms of action. Roburic acid (RA), a tetracyclic triterpenoid, has been identified as a substance capable of inhibiting key NF-κB and MAPK signaling pathways through direct interaction with TNF-α, as well as preventing the production of inflammatory mediators and cancer progression. In this study, we evaluated the biological activity of RA against a panel of human cancer cell lines—DLD-1, HT-29, and HCT-116 (colorectal), PC-3 (prostate), and BxPC-3 (pancreatic)—as well as two non-malignant lines: WI-38 (fibroblasts) and CCD-841 CoN (colon epithelium). RA exhibited a concentration-dependent inhibitory effect on cancer cell metabolic activity, with colorectal cancer cells showing relatively higher sensitivity, particularly at shorter incubation times. To distinguish between cytotoxic and cytostatic effects, we performed trypan blue exclusion combined with a cell density assessment, clonogenic assay, and BrdU incorporation assay. The results from these complementary assays confirmed that RA acts primarily through an antiproliferative mechanism rather than by inducing cytotoxicity. In addition, NF-κB reporter assays demonstrated that RA attenuates TNF-α-induced transcriptional activation at higher concentrations, supporting its proposed anti-inflammatory properties and potential to modulate pro-tumorigenic signaling. Finally, our in silico studies predicted that RA may interact with proteins such as CAII, CES1, EGFR, and PLA2G2A, implicating it in the modulation of pathways related to proliferation and cell survival. Collectively, these findings suggest that RA may serve as a promising scaffold for the development of future anticancer agents, particularly in the context of colorectal cancer.

## 1. Introduction

The clinical effectiveness of many currently used anticancer therapies is severely limited. The reason for this is the development of resistance of cells in response to the chemotherapy drugs used. Cancer cells can adapt and develop defense mechanisms, which cause a decrease in their sensitivity to drugs, especially in the case of long-term exposure. Additionally, the phenomenon of tumor heterogeneity makes some cancers more resistant due to genetic differences between cancer cells. This creates another challenge in designing uniform and effective therapies, as molecular differences between tumors may require an individualized therapeutic approach. Ultimately, damage to healthy cells leads to side effects and the deterioration of patients’ overall health. Due to the many challenges and dynamic nature of cancer diseases, there is a constant need to introduce new drugs to increase the effectiveness of and individualize treatment strategies [1].

In the search for new therapeutic agents, scientists take a variety of approaches, including computer-aided drug design (CADD) and de novo synthesis, or discovery of natural substances. New approaches based on natural substances include mainly research on phytocomponents, i.e., alkaloids, polyphenols, flavonoids, saponins, stilbene, lignans, and also compounds isolated from fungi or metabolites of marine organisms [2,3,4]. The wealth and diversity of chemical compounds present in the natural environment make them a valuable source of compounds with potential anticancer properties. Not only do they have unique chemical structures, but they also often exhibit specific, multidirectional mechanisms of action [5].

One such compound that has recently attracted the attention of researchers is roburic acid (RA). This tetracyclic triterpenoid, a terpene derivative composed of a combination of five-carbon isoprene units, has become the subject of intense research due to its diverse biological properties. Although it was probably used several centuries earlier in traditional Chinese medicine, its rediscovery occurred recently. This compound is isolated from oak galls, which are growths that result from damage to plant tissues by insects that feed on them [6,7]. Moreover, it also occurs in the plants of *Gentiana macrophylla* and *Gentiana dahurica*, growing in China, Kazakhstan, Mongolia, and Russia. It is known for its strong anti-inflammatory, antioxidant, and antimicrobial properties. Previous studies have demonstrated its ability to inhibit signaling pathways, including Nuclear factor NF-kappa-B (NF-κB) and mitogen-activated protein kinases (MAPKs), through direct interaction with Tumor necrosis factor α (TNF-α) [7,8]. Furthermore, RA may influence the regulation of the nuclear factor erythroid 2-related factor 2 (NRF2)/Kelch-like ECH-Associating protein 1 (KEAP1)/antioxidant responsive element (ARE) pathway, which contributes to the reduction of reactive oxygen species levels [9]. RA has also been classified as an inhibitor of microsomal prostaglandin E2-1 synthase, which translates into its ability to inhibit the formation of prostaglandin E2, a key mediator in inflammatory processes and a significant promoter of cancer development [10]. The first in vitro studies of this compound using cancer cells showed its ability to regulate the expression of cyclins B, D, and E or c-MYC, which results in its antiproliferative potential and antiapoptotic proteins, leading to cell death [8]. In the present study, we tested the cytotoxic/cytostatic effects of RA in normal and cancerous human cell lines in vitro and investigated the molecular basis of its anticancer activity, including its impact on inflammatory signaling. We also assessed the pharmacokinetic and pharmacodynamic properties of RA using computational modeling.

## 2. Results

### 2.1. MTT Assay

**Figure 1:**
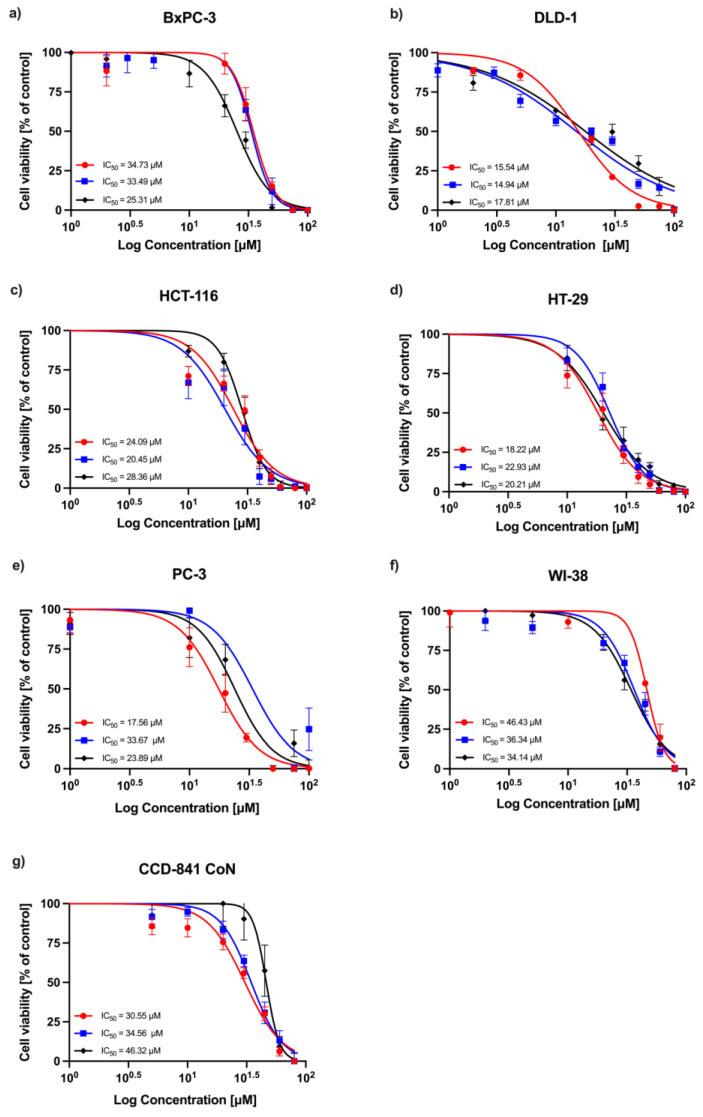
Relative cell viability (%) of cancer cell lines (**a**) BxPC-3, (**b**) DLD-1, (**c**) HCT-116, (**d**) HT-29, and (**e**) PC-3, and normal cells (**f**) WI-38 and (**g**) CCD-841 CoN after 24 h treatment with RA. The dose–response curve ± SD and the corresponding IC_50_ values are presented for each line.

**Figure 2:**
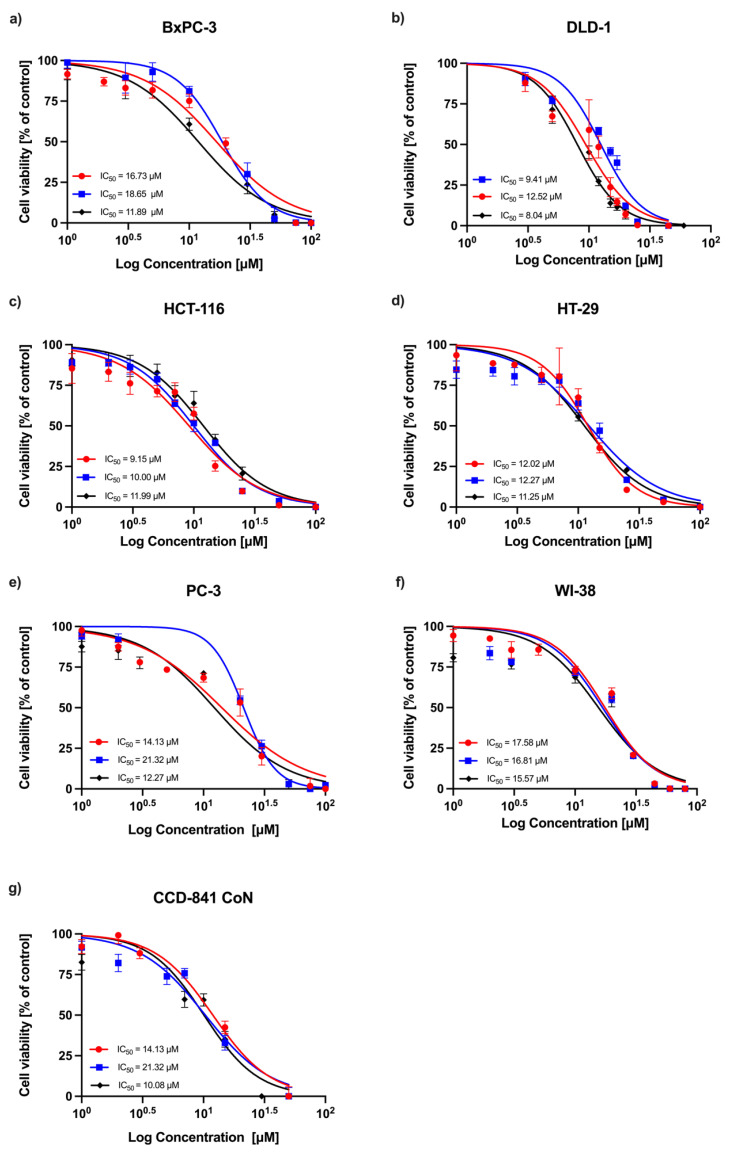
Relative cell viability (%) of cancer cell lines (**a**) BxPC-3, (**b**) DLD-1, (**c**) HCT-116, (**d**) HT-29, and (**e**) PC-3, and normal cells (**f**) WI-38 and (**g**) CCD-841 CoN after 72 h treatment with RA. The dose–response curve ± SD and the corresponding IC_50_ values are presented for each line.

A concentration-dependent decrease in absorbance was observed in all experimental series, indicating a reduction in cell viability following RA treatment. Dose–response curves obtained from three independent replicates are shown in Figure 1 (24 h incubation) and Figure 2 (72 h incubation). Among the tested cell lines, DLD-1 (colorectal adenocarcinoma) demonstrated the greatest sensitivity to RA after 24 h of incubation, followed by the HT-29 (colorectal adenocarcinoma), HCT-116 (colorectal carcinoma), PC-3 (prostatic adenocarcinoma), and BxPC-3 (pancreatic adenocarcinoma) cells. As expected, normal human fibroblasts (WI-38) and human colon epithelial cells (CCD-841 CoN) showed the lowest sensitivity to the compound. After 72 h, the sensitivity profile remained generally consistent, with DLD-1 cells exhibiting the strongest response, followed by HCT-116 and HT-29 cells. However, the IC_50_ values for the non-malignant cell lines (CCD-841 CoN and WI-38) were closer to those of the less responsive cancer cell lines (PC-3 and BxPC-3), indicating a reduced distinction in sensitivity between malignant and non-malignant cells at this timepoint. The corresponding IC_50_ values are summarized in Table 1.

### 2.2. Neutral Red Uptake Assay

Based on the results of the MTT assay, the colon cancer cell lines DLD-1, HCT-116, and HT-29, along with the normal cell lines WI-38 and CCD-841 CoN, were selected for further evaluation using the neutral red uptake assay. This test measures the ability of viable cells to incorporate neutral red dye into lysosomes, serving as an indicator of lysosomal integrity and overall cell viability. A time-dependent decrease in dye accumulation was observed following 24 and 72 h exposure to RA, with the most pronounced effect noted in the DLD-1 cell line. In contrast, the CCD-841 CoN cells exhibited the lowest sensitivity to the tested compound at both time points. The IC_50_ values calculated from two independent experiments are presented in Table 2.

### 2.3. Crystal Violet Assay

The third and final test used to assess the cellular response to RA was the crystal violet assay. The experiments were conducted in a manner analogous to the neutral red assay. The IC_50_ values obtained from two independent experiments are presented in Table 3. After 24 h, the greatest reduction in crystal violet uptake was observed in the DLD-1 cell line, followed by HCT-116, HT-29, and the normal cells WI-38 and CCD-841 CoN. However, after 72 h, the greatest reduction was observed in the HT-29 cell line, followed by DLD-1 and HCT-116. Notably, the IC_50_ value for WI-38 cells after 72 h was comparable to that of the HCT-116 cancer cells. CCD-841 CoN cells consistently exhibited the lowest response to RA treatment.

Conducting three different assays, each based on distinct mechanisms but all assessing cell viability, allowed for the determination of a unified value for each cell line, referred to as the consensus IC_50_. These values were calculated as the arithmetic mean of the results obtained from the preceding assays and are summarized in Table 4.

### 2.4. Cell Density and Viability Analysis Using Trypan Blue Exclusion

**Figure 3:**
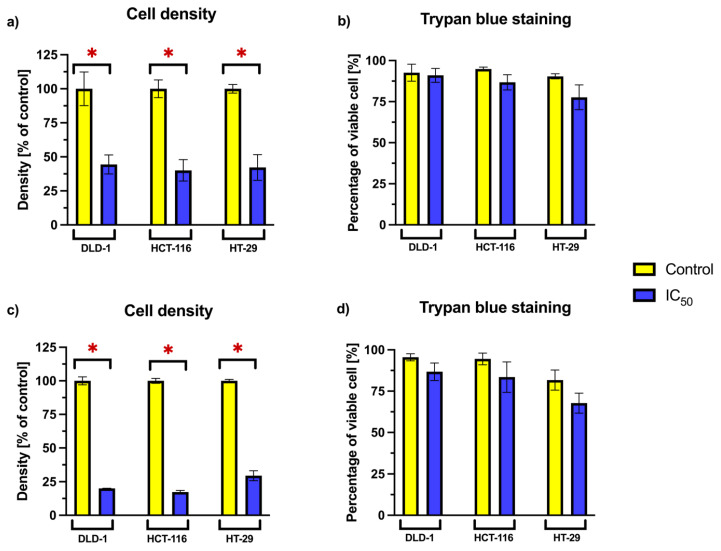
Relative cell density of colorectal cancer cell lines (DLD-1, HCT-116, and HT-29) treated with RA at consensus IC_50_ concentrations for (**a**) 24 h and (**c**) 72 h, expressed as a percentage of the untreated control. Percentage of viable cells determined by trypan blue exclusion after (**b**) 24 h and (**d**) 72 h RA treatment. Data represent mean ± SD from two independent experiments. Asterisks (*) indicate statistically significant differences compared to the control (*p* < 0.05).

To further evaluate the impact of RA on cellular proliferation and survival, we focused on the three cancer cell lines—DLD-1, HCT-116, and HT-29—that exhibited the greatest response to RA in viability assays. The relative cell density and the proportion of viable cells were analyzed after 24 and 72 h incubation periods (Figure 3). RA was applied at concentrations corresponding to the previously established consensus IC_50_ values for each cell line.

A statistically significant reduction in relative cell density was observed across all tested cell lines following RA treatment at both time points. This suggests a decrease in proliferative capacity or cell accumulation. However, the percentage of viable cells remained comparable to that of the untreated control. No statistically significant differences in viability were noted between RA-treated and untreated cells, suggesting that the reduced density was not associated with increased cell death.

### 2.5. Clonogenic Assay

**Figure 4:**
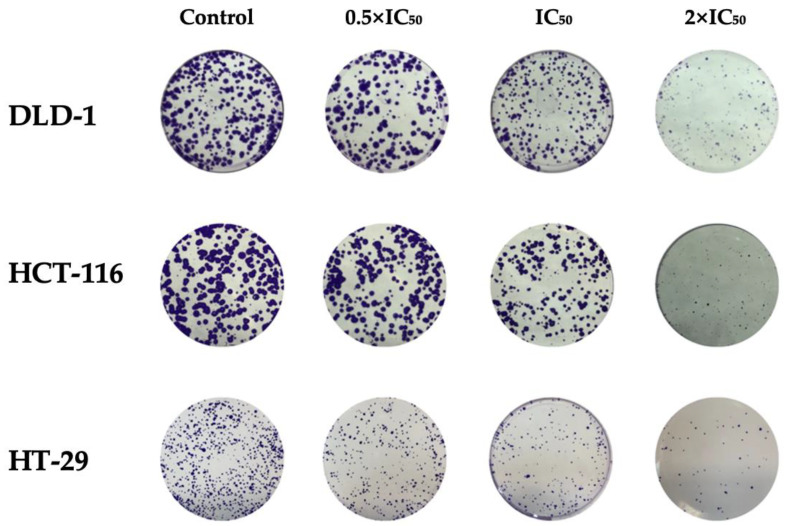
Representative images of colony formation following RA treatment. The effect of RA administered at 0.5 × IC_50_, IC_50_, and 2 × IC_50_ on the clonogenic potential of colorectal cancer cell lines DLD-1, HCT-116, and HT-29 is compared to untreated controls.

To evaluate the long-term proliferative capacity of cancer cells following RA treatment, a clonogenic assay was performed. Based on preliminary optimization, a seeding of 500 cells per well was used, which ensured consistent colony formation. Cells were then treated with RA at concentrations corresponding to 0.5 × IC_50_, IC_50_, and 2 × IC_50_ values. After a 10-day incubation period, colonies were stained and quantified (Figure 4). A statistically significant (*p* < 0.05) reduction in colony formation was observed in all tested cell lines at both IC_50_ and 2 × IC_50_ concentrations (Table 5). The number of colonies formed decreased in a dose-dependent manner. These findings indicate that RA impairs the clonogenic potential of colorectal cancer cells.

### 2.6. Bromodeoxyuridine Incorporation Assay

**Figure 5:**
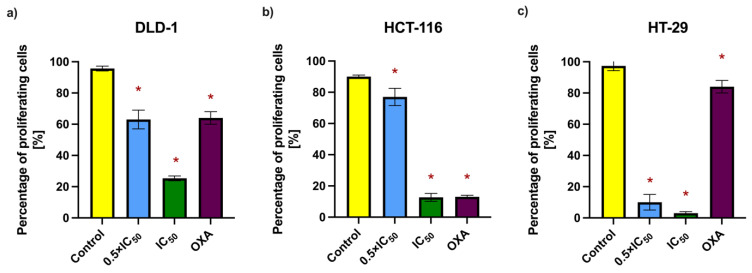
Percentage of proliferating cells in colorectal cancer cell lines (**a**) DLD-1, (**b**) HCT-116, and (**c**) HT-29 after 48 h exposure to RA at 0.5 × IC_50_ and IC_50_ concentrations, assessed by a bromodeoxyuridine incorporation assay. Data are presented as the mean percentage of proliferating cells [%] ± SD. Statistically significant differences (*p* < 0.05) compared to the negative control are marked with an asterisk (*).

To further investigate the antiproliferative potential of RA, the bromodeoxyuridine (BrdU) incorporation assay was employed. This method detects the integration of BrdU, a synthetic thymidine analog, into newly synthesized DNA during the S-phase of the cell cycle, thereby serving as a marker of active proliferation. Cells from the DLD-1, HCT-116, and HT-29 lines were incubated with RA at 0.5 × IC_50_ and IC_50_ concentrations for 48 h. Then, 15 µM oxaliplatin (OXA) was used as a positive control due to its established use in colorectal cancer therapy and well-documented antiproliferative properties. The concentration was selected based on previously reported IC_50_ values for colorectal cancer cell lines and applied uniformly across the tested models for consistency [11,12,13,14,15]. The results, expressed as the percentage of BrdU-positive cells, demonstrated a concentration-dependent decrease in proliferation following RA treatment (Figure 5 and Table 6). The most pronounced reduction was observed in the HT-29 cell line. In all tested lines, RA exhibited antiproliferative activity comparable to or exceeding that of the positive control. These findings, along with the clonogenic assay results, support the cytostatic effect of RA on colorectal cancer cells.

### 2.7. NF-κB Reporter Cell Luciferase Assay

**Figure 6:**
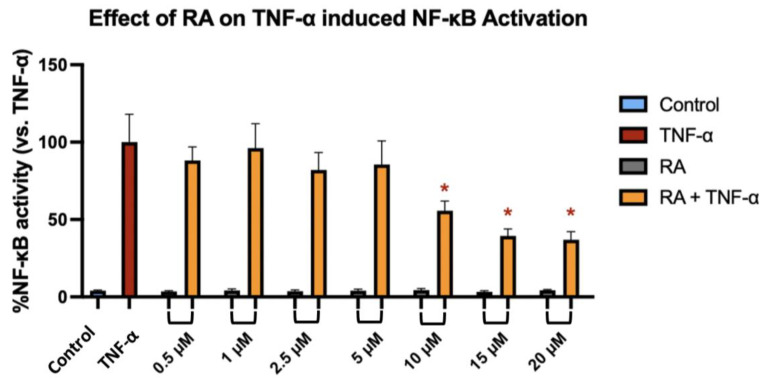
NF-κB transcriptional activity in NF-κB-TIME reporter cells following 6 h stimulation with TNF-α after 1 h pre-treatment with RA at concentrations of 0.5–20 µM. Data are presented as the mean percentage of NF-κB activity [%] relative to the TNF-α-stimulated control (set as 100%) ± SD. Statistically significant differences (*p* < 0.05) compared to the TNF-α control are marked with asterisks (*).

To investigate whether RA modulates NF-κB transcriptional activity in response to inflammatory stimulation, we used the NF-κB-TIME human endothelial reporter cell line, which contains a stably integrated luciferase construct under the control of NF-κB response elements, allowing for sensitive and quantitative monitoring of pathway activation. NF-κB-TIME cells were pre-treated with increasing concentrations of RA for 1 h, followed by 6 h stimulation with TNF-α. TNF-α alone induced a robust activation of NF-κB, as reflected by a significant increase in luminescence signal compared to the unstimulated control. RA at concentrations ranging from 0.5 to 5 µM had no statistically significant effect on NF-κB activity in TNF-α-stimulated cells. However, treatment with 10 µM RA resulted in a modest but significant reduction in NF-κB activation (*p* = 0.0399), which was further enhanced at 15 µM (*p* = 0.0036) and 20 µM (*p* = 0.0016) (Figure 6). These findings suggest that RA attenuates NF-κB signaling in a concentration-dependent manner, with measurable inhibitory effects emerging at ≥10 µM.

### 2.8. ADMET and Drug-Likeness

To assess the Absorption, Distribution, Metabolism, Excretion, and Toxicity (ADMET) characteristics and drug-likeness of RA, the Swiss-ADME and pkCSM platforms were used (http://www.swissadme.ch; https://biosig.lab.uq.edu.au/pkcsm/prediction accessed on 26 September 2023). The results are summarized in Table 7.

RA displays physicochemical and pharmacokinetic features characteristic of a lipophilic, moderately bioavailable compound. Its high lipophilicity supports its membrane permeability, but may compromise aqueous solubility and gastrointestinal absorption. Although predictive models provided mixed results, RA demonstrated good permeability in the Caco-2 model and is not a substrate for P-glycoprotein, which favors absorption [16].

The compound has a low steady state volume of distribution (VDss), suggesting high plasma protein binding and potentially reduced levels of pharmacologically active free drugs. RA is a substrate for CYP3A4, indicating possible first-pass metabolism and reduced oral bioavailability. However, it does not appear to inhibit key CYP enzymes, implying a low risk of drug–drug interactions.

The predicted total clearance is low, suggesting prolonged systemic retention, though RA is not a substrate for the renal Organic Cation Transporter 2 (OCT2). Toxicity models showed no mutagenic or cardiotoxic potential (negative AMES and hERG I/II), and the predicted maximum tolerated dose supports low acute toxicity. The long-term safety remains uncertain, particularly regarding liver effects.

Drug-likeness evaluation showed that RA violates some filters, mainly due to its high lipophilicity and structural complexity, but satisfies others, such as the Veber rule and bioavailability score. It triggered no PAINS alerts and had a moderate synthetic accessibility score.

In summary, RA appears to possess moderate toxicity and favorable bioavailability. Some challenges may arise from its complexity, potentially affecting synthesis and optimization. Nonetheless, more in-depth studies are warranted to evaluate RA’s potential as a promising scaffold for drug development.

### 2.9. Prediction of Molecular Targets and Docking Studies

The crystal structures of protein–inhibitor complexes of tyrosine phosphatase 1B (PTP1β) with 2-(oxalyl-amino)-4,7-dihydro-5H-thieno [2,3-*c*]pyran-3-carboxylic acid (PDB id: 1C87) [17], T-cell protein tyrosine phosphatase (PTPN2) with ABBV-CLS-484 (PDB id: 7UAD) [to be published], prostaglandin E synthase (PTGES) with N-[4-(4-chlorophenyl)-1H-imidazol-2-yl]-2-(difluoromethyl)-5-{[(2-methylpropanoyl)amino]methyl}benzamide (PDB id: 5BQH) [18], carboxylesterase 1 (CES1) with tamoxifen (PDB id: 1YA4) [19], fatty acid-binding protein, adipocyte with 2-[(2-phenylphenyl)amino]benzoic acid (PDB id: 6LJS), DNA-(apurinic or apyrimidinic site) endonuclease, mitochondrial (APE1) with 5-nitro-1H-indole-2-carboxylic acid (PDB id: 7TC2) [20], Cathepsin D (CPSD) with N-(3,4-dimethoxybenzyl)-N-alpha-{N-[(3,4-dimethoxyphenyl)acetyl]carbamimidoyl}-D-phenylalaninamide (PDB id: 4OD9) [21], dual specificity protein kinase CLK4 (CLK4) with 5-[(3-chlorophenyl)amino]benzo[c][2,6]naphthyridine-8-carboxylic acid (PDB id: 6FYV) [22], glycogen phosphorylase (PYGL) with N-(2-chloro-4-fluorobenzoyl)-N’-(5-hydroxy-2-methoxyphenyl)urea (PDB id: 2ATI) [23], phospholipase A2 (PLA2G2A) with 6-phenyl-4(R)-(7-phenyl-heptanoylamino)-hexanoic acid (PDB id: 1KQU) [24], carbonic anhydrase 2 (CAII) with acetazolamide (PDB id: 3HS4) [25], and epidermal growth factor receptor (EGFR) with erlotinib (PDB id: 1M17) [26] were obtained from the PDB database (https://www.rcsb.org/), accessed on 25 September 2023. To validate the docking procedure used here, we have re-docked the co-crystallized ligands into the same binding site as described in the PDB files previously procured. Following the validation of the docking procedure, it was used to dock RA into the active site of the predicted targets (Table S1 of the Supplementary Information). It was shown that RA complexed with CAII, CES1, EGFR, and PLA2G2A showed the best binding score, with competitive binding affinity to reference inhibitors reported in the literature and deposited in the PDB database, and complexed with the protein target structures. The two-dimensional and three-dimensional representations of RA interactions within the active sites of these targets are shown in Figures S1–S13 of the Supplementary Information.

### 2.10. Molecular Dynamics Simulation

RA complexed with each of the CAII, CES1, EGFR, and PLA2G2A receptors were shortlisted for performing molecular dynamics (MD) simulation based on their docking score and observed drug–receptor chemical interactions.

A drug–receptor complex has to be sufficiently stable over a nano-scale time range to execute its therapeutic response. The complex of the CAII enzyme with RA was evaluated for thermodynamic stability by MD simulation for a specified timeframe of 100 ns by using Schrodinger’s Desmond software version 2022.4. The CAII enzyme’s monomeric chain has 257 amino acids consisting of 2049 heavy atoms out of an overall 4055 atoms. Structural alterations and root-mean-square deviation (RMSD) analysis of the macromolecular backbone were executed during the 100 ns simulation to evaluate their thermodynamic stability. The complexed ligand RA constitutes five flexible bonds comprising thirty-two heavy atoms of eighty atoms in total. CAII conjugated with RA displayed that the bound ligand showed a couple of conformational changes at 20 ns and 75 ns, followed by attaining stabilized conformation. The RMSD value of the receptor’s backbone was found to fluctuate between 0.8 and 1.6 Å, whereas the bound ligand RA exhibited minor fluctuations after achieving stabilized conformation at 75 ns and its RMSD value in the receptor cavity fluctuated between 7.0 and 9.0 Å.

The CES1 receptor’s monomeric chain has 531 amino acids consisting of 4124 heavy atoms out of an overall 8257 atoms. The CES1 receptor conjugated with ligand RA displayed that the bound ligand showed some fluctuations until the initial 40 ns of simulation, followed by attaining stabilized conformation. The RMSD value of the receptor’s backbone was found to fluctuate between 1.00 and 1.75 Å, whereas the bound ligand RA was found to fluctuate between 2.0 and 4.5 Å.

The EGFR receptor’s monomeric chain has 312 amino acids consisting of 2497 heavy atoms out of an overall 5031 atoms. The EGFR conjugated with ligand RA was found to be stabilized throughout the simulation, with the RMSD value of the receptor’s backbone found to fluctuate between 6.5 and 10.5 Å, whereas the bound ligand RA exhibited very minute fluctuations and maintained stabilized conformation throughout the simulation with an RMSD value in the receptor cavity between 4.5 and 6.5 Å.

The PLA2G2A receptor’s monomeric chain has 124 amino acids consisting of 942 heavy atoms out of an overall 1834 atoms. The PLA2G2A receptor conjugated with ligand RA displayed that the bound ligand showed a high degree of stability during the simulation. The RMSD value of the receptor’s backbone was found to fluctuate between 1.5 and 3.5 Å, whereas the bound ligand RA exhibited high stability in its RMSD value within the receptor cavity, ranging from 3.5 to 7.5 Å. The RMSD profiles of RA conjugated with CAII, CES1, EGFR, and PLA2G2A are presented in Figure S14 (Supplementary Information).

The atoms in a protein or ligand structure might deviate from their initial location, and this can be measured by using their root-mean-square fluctuation (RMSF) value. This is an important parameter for determining the flexibility and dynamic behavior of the macromolecular complex. Protein RMSF is important because it may be used to predict protein dynamics and evaluate stability by providing information about the relative flexibility of various regions. MD-based evaluation of ligand RA complexed with CAII has concluded that the RMSF for Cα backbone was found to be within 0.1–1.6 Å, while for ligand RA it was found to range from 2.0 to 5.5 Å. The RMSF for ligand RA complexed with CES1 was found to be within 0.5–2.5 Å for the Cα backbone, while for the complexed ligand RA, the average variation was found to be within 1.0–2.5 Å. The RMSF for ligand RA complexed with EGFR was found to be within 1.5–4.5 Å for the Cα backbone, except for some terminal residues, while the complexed ligand RA’s average variation was found to be within 2.0–4.5 Å. The RMSF for the PLA2G2A enzyme Cα backbone complexed with ligand RA was revealed to be within 0.8–3.0 Å for the protein Cα backbone, while the ligand RA’s average variation was found to be within 2.0–3.0 Å. Figures S15–S18 (Supplementary Information) show the RMSF profiles of the Cα backbones and bound RA ligands for CAII, CES1, EGFR, and PLA2G2A, respectively, as detected during MD simulations.

The development of hydrophobic contacts, ionic interactions, and hydrogen bonds during an MD simulation are responsible for the thermodynamic permanence of a receptor–ligand complex, and it is evaluated by the continuous monitoring of its strength throughout the simulation for all three macromolecular complexes. The simulation of the CAII receptor complexed with ligand RA was found to be interacting with the CAII protein via the formation of hydrophobic bonds with the amino acids Trp5, Leu60, Val121, Phe131, Val135, Leu141, Leu198, Pro202, Leu204, and Trp209, with Asn62, His96, Gln136, and Thr200 via hydrogen bonds, and the amino acids Trp5, Tyr7, His64, Asn67, Gln92, Gly132, Gln136, Thr200, and Pro201 are found to be interacting via water bridges. In another simulation, the CES1 protein complexed with ligand RA was found to be interacting with the CES1 protein via the formation of hydrophobic bonds with the amino acids Ala93, Leu96, Leu97, Leu100, Phe101, Val146, Val254, Leu255, Leu304, Pro317, Leu318, Ile359, Leu363, Met364, Leu388, Met425, and Phe426, with Leu304 and Leu306 via hydrogen bonds, and the amino acids Leu304, Leu306, Pro317, Leu363, and Met364 are found to be interacting via water bridges. In another simulation, the EGFR receptor complexed with ligand RA was found to be interacting with the EGFR receptor via the formation of hydrophobic bonds with the amino acids Leu694, Phe699, Val702, Ala719, and Leu820, with Thr766, Gln767, and Met769 via hydrogen bonds, and the amino acids Thr766, Gln767, Met769, Phe771, and Thr830 were found to be interacting via water bridges. In another simulation, the PLA2G2A receptor complexed with ligand RA was found to be interacting with the PLA2G2A receptor via the formation of hydrophobic bonds with the amino acids Leu2, Phe5, Ile9, Ala17, Ala18, Tyr21, Tyr51, and Phe98, with His27, Gly29, Val30, and Gly31 via hydrogen bonds, and the amino acids His27, Gly29, Val30, Gly31, Gly32, Cys44, His47, and Asp48 were found to be interacting via water bridges. Figure S19 (Supplementary Information) illustrates the chemical interactions between RA and the binding site residues of the (a) CAII, (b) CES1, (c) EGFR, and (d) PLA2G2A receptors.

## 3. Discussion

Natural compounds derived from plants have long served as valuable sources of bioactive agents in cancer therapy. Among these, RA, a triterpenoid compound isolated from various *Gentiana* species, has recently gained attention due to its anti-inflammatory and potential anticancer properties. However, despite its promising biological profile, RA remains relatively understudied in the context of oncology, particularly regarding its activity in solid tumors and the mechanisms underlying its effects on cancer and normal cells.

Previous studies have indicated that RA may exert antiproliferative and proapoptotic effects in certain cancer models. These effects have been associated with cell cycle arrest at the G0/G1 phase, potentially mediated by downregulation of cyclins B1, D1, and E1, as well as with increased apoptotic signaling evidenced by poly(ADP-ribose) polymerase-1 (PARP1) cleavage and the activation of caspases 3, 7, and 9 [8]. Mechanistic studies suggest that RA may exert its activity through direct interaction with TNF-α, thereby inhibiting the NF-κB pathway [8]. Additionally, RA has been reported to possess anti-inflammatory properties through inhibition of cyclooxygenase enzymes and by competitively binding to tumor necrosis factor receptor 1 (TNF-R1), further contributing to its proapoptotic potential [27]. In colorectal cancer cells, RA treatment has been shown to inhibit DNA synthesis, as demonstrated by 5-ethynyl-2′-deoxyuridine (EdU) incorporation assays, and to induce morphological changes including cell shrinkage, detachment from colonies, and cell death [8]. Apoptosis induction has been further confirmed by annexin V staining and flow cytometric analysis [8]. However, despite these promising findings, the current body of evidence remains fragmented, often limited to a single cell line or narrowly focused on individual pathways, and lacks systematic evaluation across different cancer types or normal cell counterparts. In particular, the distinction between the cytotoxic and cytostatic effects of RA has not been clearly established. Therefore, further studies are needed to comprehensively assess the biological activity of RA, to determine whether its observed effects are primarily due to cell death or proliferation arrest, and to evaluate its selectivity across both malignant and non-malignant human cell lines.

In the present study, we evaluated the biological activity of RA against a panel of human cancer cell lines derived from different tissue origins, including DLD-1 and HT-29 (colorectal adenocarcinoma), HCT-116 (colorectal carcinoma), PC-3 (prostate adenocarcinoma), and BxPC-3 (pancreatic adenocarcinoma). To assess selectivity, we also included two non-malignant cell lines: WI-38 (normal human fibroblasts) and CCD-841 CoN (normal human colon epithelial cells). Cell viability and metabolic activity were assessed using MTT, neutral red uptake, and crystal violet assays following 24 and 72 h incubations with RA. Across all three assays, RA induced a concentration-dependent reduction in signal intensity, which initially suggested either decreased viability or inhibited proliferation. Cancer cell lines were more responsive, with IC_50_ values ranging from 14.16 to 31.18 µM after 24 h and from 8.25 to 15.91 µM after 72 h. In contrast, non-tumorigenic cells displayed higher IC_50_ values at 24 h (33.13–38.97 µM), supporting a favorable selectivity profile at early timepoints. However, after 72 h of incubation, this distinction became less evident, as the IC_50_ values for normal cells (12.82–19.34 µM) approached those of the less responsive cancer cell lines (e.g., BxPC-3 and PC-3). This observation suggests that the compound’s selectivity decreases with prolonged exposure. Despite this overall pattern, colorectal cancer cell lines tended to respond more strongly to RA than the other tumor types. Based on these observations, DLD-1, HCT-116, and HT-29 cells were selected for subsequent mechanistic studies.

To further elucidate whether the observed decrease in metabolic activity was due to cytotoxicity or reduced proliferation, we employed three complementary approaches: a cell density analysis combined with trypan blue exclusion, clonogenic assay, and BrdU incorporation assay. These experiments were conducted using DLD-1, HCT-116, and HT-29 cells, selected for their pronounced response to RA in prior viability assessments. Initial evidence for a cytostatic effect came from cell density assessments, which revealed a marked reduction in cell confluence following RA treatment without a significant decrease in viability, as determined by trypan blue staining. Additional confirmation was provided by the clonogenic assay, which demonstrated a significant reduction in the number of colonies formed by DLD-1, HCT-116, and HT-29 cells treated with RA at both IC_50_ and 2 × IC_50_ concentrations. Finally, the BrdU incorporation assay confirmed that RA inhibits DNA synthesis, thereby substantiating its antiproliferative mechanism of action. Notably, in the HT-29 cell line, RA was more effective at suppressing BrdU incorporation than OXA, a chemotherapeutic agent used as a positive control. Collectively, these findings suggest that RA exerts its anticancer effects primarily by suppressing cellular proliferation rather than inducing direct cytotoxicity. The diminished selectivity observed after prolonged exposure is likely a consequence of this antiproliferative mode of action, which, over time, also affects slower-dividing non-malignant cells. Unlike many classical chemotherapeutics, which rely on acute cytotoxic mechanisms, RA’s ability to reduce proliferation without causing significant cell death may offer a more favorable safety profile. This characteristic positions RA as a potential candidate for combination therapies or for use in treatment regimens requiring lower systemic toxicity.

Beyond its effect on cancer cell proliferation, we investigated its potential to modulate NF-κB signaling, a pathway critically involved in inflammation-driven tumor progression, resistance to apoptosis, and therapy failure in various cancers [28,29]. To this end, we used the NF-κB-TIME reporter cell line, which harbors a stably integrated NF-κB-responsive luciferase construct and provides a highly sensitive and reproducible model for monitoring transcriptional responses to inflammatory stimuli such as TNF-α. This system enables direct quantification of pathway activation without reliance on downstream target genes, making it particularly suited for pharmacological screening. Since this cell line is not tumor-derived and is optimized for transcriptional signaling rather than viability assays, it was not included in cytotoxicity or proliferation studies such as MTT or BrdU. We aimed to functionally verify previous observations suggesting the anti-inflammatory properties of RA by determining its ability to interfere with TNF-α-induced NF-κB activity [7,8]. Our results demonstrated that RA significantly reduced NF-κB-mediated reporter expression at concentrations of 10 µM, 15 µM, and 20 µM, even after short-term exposure (6 h total incubation and 1 h pre-treatment). Lower concentrations had no detectable effect. The compound was tested across a wide concentration range (0.5–20 µM) to capture potential transcriptional effects. As the assay was not intended to assess cell viability, IC_50_ values were not determined. Instead, fixed concentrations were selected based on prior results to avoid concentrations that significantly affect cell growth, ensuring a reliable assessment of NF-κB modulation. These findings indicate that RA attenuates inflammatory signaling in a concentration-dependent manner, reinforcing the view that its biological effects may extend beyond proliferation control to include suppression of pro-tumorigenic signaling networks. This observation is particularly relevant given that NF-κB is frequently hyperactivated in colorectal cancer and contributes to a microenvironment that supports tumor growth and immune evasion [30,31]. Therefore, the ability of RA to modulate this pathway may enhance its therapeutic profile and support its further evaluation as a candidate for inflammation-associated malignancies.

Additionally, we performed in silico screens to investigate RA as a scaffold for future drug development. Computational predictions indicated that RA may exhibit mild toxicity and satisfactory oral bioavailability, although its complex chemical structure could pose challenges for synthesis and optimization. Molecular docking and dynamics simulations suggested that RA may exert its anticancer effects through interactions with several proteins involved in cell proliferation and death, including CAII, CES1, EGFR, and PLA2G2A. While CAII, EGFR, and PLA2G2A are associated with various malignancies, CES1 showed the most stable interaction with RA in our simulations, with low RMSD and RMSF values and persistent binding within the active site. CES1 is a metabolic lipase regulated by NF-κB and is known to support fatty acid oxidation and energy balance in aggressive colorectal cancers. Its overactivity contributes to cancer cell survival by preventing the accumulation of cytotoxic lipid species [32]. Previous studies have shown that pharmacological or genetic inhibition of CES1 alters lipid metabolism, compromises mitochondrial function, and sensitizes tumor cells to chemotherapeutic agents. Notably, targeting the CES1/PPARα/stearoyl-CoA desaturase 1 pathway has been reported to enhance the efficacy of cisplatin in hepatocellular carcinoma models, with coadministration reducing tumor growth in xenografted mice [33]. Based on these computational findings and the biological relevance of CES1 in cancer, we propose that CES1 may represent a promising molecular target for RA and its derivatives. However, it should be emphasized that our docking and simulation results are predictive in nature and serve as a preliminary hypothesis-generating framework that awaits experimental confirmation.

## 4. Materials and Methods

### 4.1. Cell Culture

Human cancer cell lines BxPC-3 (pancreatic adenocarcinoma, ATCC^®^ CRL-1687^TM^), DLD-1 (colorectal adenocarcinoma, ATCC^®^ CCL-221^TM^), HCT-116 (colorectal carcinoma, ATCC^®^ CCL-247^TM^), HT-29 (colorectal adenocarcinoma, ATCC^®^ HTB-38^TM^), and PC-3 (prostate cancer, ATCC^®^ CRL-1435TM), as well as normal cell lines WI-38 (human lung fibroblasts, ATCC^®^ CCL-75^TM^) and CCD-841 CoN (human colon epithelial, ATCC^®^ CRL-1790^TM^) were obtained from the American Type Culture Collection (ATCC, Rockville, MD, USA). In addition, the NF-κB-TIME reporter cell line (human endothelial origin, ATCC^®^ CRL-4049™), which carries a luciferase reporter under the control of NF-κB response elements, was kindly provided by the Institute of Vascular Biology and Thrombosis Research at the Medical University of Vienna, Vienna, Austria. BxPC-3, DLD-1, HCT-116, and HT-29 cells were cultured in RPMI-1640 medium; PC-3 cells in DMEM/F12; WI-38 and CCD-841 CoN in MEM Alpha medium; and NF-κB-TIME cells in vascular cell basal medium supplemented with a bovine brain extract growth kit, 12.5 μg/mL blasticidin, and 20 μg/mL hygromycin B. All media were further supplemented with 10% (*v*/*v*) fetal bovine serum (FBS) and 1% (*v*/*v*) streptomycin–penicillin solution.

Cells were maintained at 37 °C in a humidified atmosphere of 5% CO_2_. The culture medium was refreshed every 24–48 h. After the cells reached confluence, subculture was performed using 0.25% trypsin-EDTA. Mycoplasma contamination was routinely monitored using the MycoBlue^TM^ Mycoplasma Detector kit (Vazyme BiotechCo., Ltd., Nanjing, China).

### 4.2. MTT Assay

To evaluate the effect of the test compound on cell viability, the MTT assay was employed. This colorimetric assay measures the metabolic activity of cells, based on their ability to reduce the yellow tetrazolium salt to insoluble violet-blue formazan crystals via mitochondrial succinate dehydrogenase. The amount of formazan produced is directly proportional to the number of metabolically active (viable) cells. After solubilization of the formazan crystals in an organic solvent, absorbance can be measured spectrophotometrically [34]. Cells (8–10 × 10^3^/well) were seeded in 96-well plates and incubated for 24 h (37 °C, 5% CO_2_), then treated with RA (0.5–100 µM) for 24 or 72 h. After incubation, 20 µL of MTT solution (5 mg/mL) was added per well and incubated for 3 h. Formazan crystals were dissolved in 100 µL of dimethyl sulfoxide (DMSO), and absorbance was measured at 570 nm using a microplate reader (Power Wave XS, BioTek Instruments, Winooski, VT, USA). The IC_50_ value—the concentration of RA required to reduce cell viability by 50%—was calculated based on absorbance values using non-linear regression analysis in GraphPad Prism 8.0 (GraphPad Software, San Diego, CA, USA) [35].

### 4.3. Neutral Red Uptake Assay

Cells were seeded in 96-well plates following the same procedure as described for the MTT assay. After compound exposure, cells were incubated with 100 µL of neutral red solution (40 µg/mL) for 2 h. After washing with phosphate-buffered saline (PBS), 150 µL of destaining solution (50% ethanol, 49% water, and 1% glacial acetic acid) was added, and plates were shaken for 15 min. Absorbance was measured at 540 nm using a microplate reader. The procedure was adapted from the protocol described by Kciuk et al. [36]. The GraphPad Prism 8.0 software was used to calculate the IC_50_ values.

### 4.4. Crystal Violet Assay

Cells were seeded analogously to the previously described methods. After incubation, cells were washed with PBS and fixed with 75% methanol (100 µL/well, 20 min, 4 °C), followed by 100% methanol (20 min, 4 °C). Fixed cells were stained with 0.2% crystal violet (100 µL/well, 20 min, RT), washed with water, dried, and the dye was solubilized in DMSO. Absorbance was measured at 590 nm using a microplate reader. IC_50_ values were determined using the GraphPad Prism 8.0 software.

### 4.5. Cell Density and Viability Analysis Using Trypan Blue Exclusion

To evaluate the mechanism of RA action, cell density and viability were assessed after 24 and 72 h incubations with RA at the consensus IC_50_. Culture medium from each well was collected, and cells were washed with PBS and detached using trypsin-EDTA. After neutralization with complete medium, the total cell number was determined using a Thoma counting chamber and expressed as a percentage relative to untreated controls. In parallel, cell viability was assessed using the trypan blue exclusion method. A 1:1 mixture of cell suspension and 0.4% trypan blue was prepared, and the viability was calculated as the percentage of unstained (viable) cells among a minimum of 300 counted.

### 4.6. Clonogenic Assay

To evaluate clonogenic potential, cells were first seeded at varying densities (100–2000 cells/well) in 6-well plates. The highest plating efficiency was observed at 500 cells/well, which was used for further experiments. After cell attachment (2–3 h), RA was added at 0.5 × IC_50_, IC_50_, and 2 × IC_50_ concentrations. Cells were incubated for 10 days, with media replaced every 3 days. Colonies were then fixed with 6% formaldehyde (10 min), stained with crystal violet (10 min), washed, dried, and imaged. Colonies were counted using the ImageJ software, version 1.54f.

### 4.7. Bromodeoxyuridine Incorporation Assay

The BrdU incorporation assay was performed to assess RA’s effect on DNA synthesis. Cells were seeded onto sterile round coverslips placed in 12-well plates at a density of 30,000 cells per well. After 24 h, cells were treated with RA at 0.5 × IC_50_ and IC_50_ concentrations for 48 h. OXA (15 µM) served as a positive control, with the concentration selected based on previously reported IC_50_ values for colorectal cancer cell lines to ensure standardized and pharmacologically relevant conditions [11,12,13,15]. BrdU was added to the culture medium at a final concentration of 10 µM, 24 h prior to fixation. Following treatment, cells were fixed with 70% ethanol, and DNA was denatured using 4N HCl. After neutralization with 0.1 M borax, cells were incubated with a primary anti-BrdU antibody (1:100, Purified Mouse Anti-BrdU by BD™), followed by a secondary Alexa Fluor 488-conjugated anti-mouse antibody (1:650, Alexa Fluor 488 goat anti-mouse IgG (H + L) Invitrogen by Thermo Fisher Scientific). Nuclei were counterstained with DAPI. Coverslips were mounted using Fluoromount-G and examined under a fluorescence microscope (Olympus, Tokyo, Japan, CellSens V2.3 software). BrdU-positive cells were quantified based on green fluorescence, counting ~200 cells per sample.

### 4.8. NF-κB Reporter Cell Luciferase Assay

To evaluate the effect of RA on TNF-α-induced NF-κB transcriptional activity, we employed the NF-κB-TIME luciferase reporter assay. Reporter cells were seeded into white-walled 96-well plates (Thermo Fisher Scientific, Waltham, MA, USA) at a density of approximately 10,000 cells per well and incubated overnight under standard conditions. On the following day, cells were pre-treated with RA (0.5–20 μM) for 1 h, followed by co-stimulation with TNF-α (10 ng/mL; PeproTech, London, UK) for an additional 6 h. NF-κB activity was then quantified by luminescence measurement using the Nano-Glo^®^ Luciferase Assay System (Promega, Madison, WI, USA) and recorded in relative light units with a TriStar^2^ S LB 942 plate reader (Berthold Technologies, Bad Wildbad, Germany). The concentration range was chosen to avoid the antiproliferative effects observed at higher doses, enabling the interpretation of transcriptional responses independent of growth inhibition. IC_50_ values were not determined, as the assay was not designed to assess cytotoxicity.

### 4.9. ADMET and Drug-Likeness Analyses

ADMET profiling and drug-likeness assessments are crucial for evaluating the suitability of chemical compounds as potential drug candidates. ADMET analysis provides insight into the absorption, distribution, metabolism, excretion, and potential toxicity of a compound, helping to predict its behavior in biological systems. In parallel, drug-likeness evaluation determines whether a compound exhibits physicochemical and structural features typical of known drugs, thereby aiding in the selection of candidates with promising pharmacokinetic and pharmacodynamic properties. In this study, the ADMET characteristics and drug-likeness of RA were evaluated using two publicly available in silico tools: Swiss-ADME (http://www.swissadme.ch) (accessed on 26 September 2023) and pkCSM (https://biosig.lab.uq.edu.au/pkcsm/prediction) (accessed on 26 September 2023).

### 4.10. Prediction of Molecular Targets and Docking Studies

The PDB databank (https://www.rcsb.org/), accessed on 25 September 2023, was checked for possible molecular targets of RA previously predicted based on the isomeric SMILES code obtained from the PubChem database (https://pubchem.ncbi.nlm.nih.gov/compound/Roburic-acid (accessed on 25 September 2023)) with the use of SwissTargetPrediction (http://www.swisstargetprediction.ch/ (accessed on 25 September 2023)), SuperPred3 (https://prediction.charite.de/ (accessed on 25 September 2023)), and BindingDB ((https://www.bindingdb.org/ (accessed on 25 September 2023)) for ligand similarity of at least 0.8) webservers. Their involvement in cancer pathogenesis was confirmed through the mining of the PubMed database (https://pubmed.ncbi.nlm.nih.gov/ (accessed on 25 September 2023)) using the following queries: “cancer” AND “protein name”.

The structure of RA was created using the ChemDraw 8.0 software, and its three-dimensional arrangement was obtained by performing energy minimization using an MM2 force field. Before conducting the molecular docking simulation, we utilized AutoDock 4, a software developed by The Scripps Research Institute in La Jolla, CA, USA. This software was used to detect aromatic carbons and rotatable bonds, establish automatic torsion parameters, consider non-polar hydrogens, and add Gasteiger charges. The AutoDock 4 tools were employed to assign autodock atom types (AD4) to the macromolecules, and Gasteiger charges were integrated and distributed throughout the macromolecule. Subsequently, the structures were stored in the PDBQT file format. To validate the process of molecular docking simulation, the structure representations of all the produced macromolecular targets were first docked with their respective reference ligands, which were already in complex structural models.

### 4.11. Molecular Dynamics Simulations

RA complexed with the CAII, CES1, EGFR, and PLA2G2A proteins were shortlisted for performing MD simulation based on their docking score and observed drug–receptor chemical interactions. MD simulation was executed for each of the above-mentioned macromolecular complexes for a time of 100 ns by using the Desmond module of Schrodinger’s Maestro software version 2022.1 [37,38,39]. The addition of explicit solvent molecules was followed by their neutralization by adding the respective ions. The steepest-descent algorithm was used to relax the system and eliminate any steric clashes or poor contacts within atoms to minimize the system’s energy. Using a short series having a low temperature with constant pressure simulations, the system was brought to equilibrium. Positional constraints are applied to the system in addition to a progressive increase in temperature [40,41,42]. This makes it more likely that the system will be in a stable, balanced state before the simulation; to obtain the appropriate outcomes, the simulation is performed for 100 ns while taking into account the system’s energies, atom positions, and RMSD values. This aids in comprehending the system’s dynamic behavior and provides long-term intuitions on the complex’s structure and functional stability [43,44].

### 4.12. Statistical Analysis

Statistical analysis was performed using the GraphPad Prism 10.0 software (GraphPad Prism Software Inc., San Diego, CA, USA). The results obtained in the MTT test came from 3 independent experiments and were presented as mean values with standard deviations (SDs). The outcomes of the neutral red uptake assay and the crystal violet assay, as complementary methods, were performed in duplicate. All results were transformed by using the logarithmic function log (X), where X was the concentration used. The next step was to normalize the results, and finally, plot a response–inhibition curve using a non-linear regression model. The use of these tools made it possible to determine the IC_50_ parameter values. For cell density and viability data obtained via the trypan blue exclusion assay, statistical significance between the treated and control groups was evaluated using the unpaired two-tailed Student’s t-test. Prior to analysis, the assumption of normal distribution was verified using the Shapiro–Wilk test. For the clonogenic assay and BrdU incorporation assay, differences among multiple treatment groups were assessed using one-way analysis of variance (ANOVA), followed by Tukey’s post hoc test for pairwise comparisons. In the NF-κB reporter assay, luminescence values were normalized to the TNF-α-stimulated control group, and statistical comparisons were performed using one-way ANOVA with Dunnett’s post hoc test. A *p*-value of less than 0.05 was considered statistically significant in all analyses.

## 5. Conclusions

RA is a plant-derived compound primarily known for its anti-inflammatory properties, but recent studies have highlighted its potential as an anticancer agent. In this study, we demonstrated that RA reduces metabolic activity, inhibits colony formation, and decreases DNA synthesis in various cancer cell lines, with colorectal models (DLD-1, HCT-116, and HT-29) showing relatively greater sensitivity, particularly at shorter incubation times. These findings support an antiproliferative rather than cytotoxic mode of action. In addition, we showed that RA significantly attenuates TNF-α-induced NF-κB activation in a reporter assay, indicating its ability to modulate inflammatory signaling pathways commonly involved in tumor progression. Furthermore, computational studies suggest that RA may exert its therapeutic potential through interaction with the CES1 receptor, as the macromolecular complex of CES1-RA was found to be highly stabilized throughout the simulation time, with a very low RMSD and RMSF value for the Cα chain as well as complexed RA, with strong interactions with the macromolecular residues throughout the simulation. In particular, CES1 may represent an interesting molecular target for RA or its derivatives in the context of anticancer therapy for aggressive colorectal cancers.

## Figures and Tables

**Table 1:** Mean IC_50_ values (± SD) obtained after 24 h and 72 h incubation of the tested cell lines with RA, as determined by the MTT assay.

Time	Cell Lines						
	Mean IC_50_ [µM] of Roburic Acid ± SD						
	BxPC-3	DLD-1	HCT-116	HT-29	PC-3	WI-38	CCD-841 CoN
24 h	31.18 ± 5.12	16.1 ± 1.51	24.3 ± 4.0	20.45 ± 2.36	25.04 ± 8.12	38.97 ± 6.55	37.14 ± 8.2
72 h	15.76 ± 3.48	9.99 ± 2.3	10.38 ± 1.46	11.85 ± 0.53	15.91 ± 4.78	16.65 ± 1.01	15.18 ± 5.69

**Table 2:** Mean IC_50_ values (± SD) obtained after 24 h and 72 h incubation of the tested cell lines with RA in the neutral red uptake assay.

Time	Cell Lines				
	Mean IC_50_ [µM] of Roburic Acid ± SD				
	DLD-1	HCT-116	HT-29	WI-38	CCD-841 CoN
24 h	14.16 ± 1.37	17.83 ± 1.62	21.57 ± 5.25	33.53 ± 1.05	33.95 ± 0.76
72 h	7.43 ± 1.58	12.86 ± 3.06	11.69 ± 1.13	16.46 ± 0.62	19.34 ± 0.02

**Table 3:** Mean IC_50_ values (± SD) obtained after 24 h and 72 h incubation of the tested cell lines with RA in the crystal violet assay.

Time	Cell Lines				
	Mean IC_50_ [µM] of Roburic Acid ± SD				
	DLD-1	HCT-116	HT-29	WI-38	CCD-841 CoN
24 h	18.45 ± 0.42	18.88 ± 0.96	25.15 ± 1.27	33.14 ± 2.08	34.65 ± 0.28
72 h	10.2 ± 1.34	12.94 ± 0.37	8.25 ± 0.64	12.82 ± 1.25	17.99 ± 0.74

**Table 4:** The consensus IC_50_ values (± SD), obtained after 24 h and 72 h incubation of the tested cell lines with RA, based on results from the MTT, neutral red uptake, and crystal violet assays.

Time	Cell Lines				
	Mean IC_50_ [µM] of Roburic Acid ± SD				
	DLD-1	HCT-116	HT-29	WI-38	CCD-841 CoN
24 h	16.23 ± 1.1	20.33 ± 2.18	22.39 ± 2.96	35.21 ± 3.23	35.25 ± 3.08
72 h	9.21 ± 1.74	12.06 ± 1.63	10.6 ± 0.77	15.32 ± 0.96	17.5 ± 2.15

**Table 5:** Mean number of colonies formed by cancer cells ± SD. Statistically significant differences compared to the negative control are indicated in bold.

RA Concentration	Cell Lines		
	Mean Number of Colonies ± SD		
	DLD-1	HCT-116	HT-29
Control	215.5 ± 21.92	244.0 ± 15.56	150.5 ± 7.78
0.5 × IC_50_	180.0 ± 15.56	168.5 ± 36.06	133.5 ± 6.36
IC_50_	**123.0 ± 7.07**	**127.0 ± 15.56**	**121.0 ± 4.24**
2 × IC_50_	**76.5 ± 6.36**	**76.0 ± 18.38**	**109.0 ± 1.41**

**Table 6:** Mean percentage [%] ± SD of proliferating cells in the populations of (a) DLD-1, (b) HCT-116, and (c) HT-29 cell lines after 48 h incubation with RA at 0.5 × IC_50_ and IC_50_ concentrations.

Cell Lines	Mean of proliferating cells [%] ± SD			
	Control	0.5 × IC_50_	IC_50_	OXA (15 µM)
DLD-1	95.5 ± 2.12	**66.0 ± 4.24**	**25.5 ± 2.12**	**66.0 ± 2.83**
HCT-116	90.5 ± 0.71	**79.5 ± 4.95**	**11.5 ± 2.12**	**12.5 ± 0.71**
HT-29	97.33 ± 3.06	**10.0 ± 5.0**	**3.0 ± 1.0**	**84.0 ± 4.0**

**Bold** values indicate statistically significant differences (p < 0.05) compared to the negative control.

**Table 7:** Predicted ADMET properties of RA obtained using the Swiss-ADME and pkCSM webservers.

Property	Prediction	Unit/Category
Absorption		
Water solubility	−4.485	log mol/L
CaCo-2 permeability	1.291	log Papp in 10^−6^ cm/s
Intestinal absorption (human)	96.428	% absorbed
Skin permeability	−2.55	log Kp
P-glycoprotein substrate	No	Yes/No
P-glycoprotein I inhibitor	No	Yes/No
P-glycoprotein II inhibitor	Yes	Yes/No
Distribution		
VDss (human)	−0.894	Log L/kg
Fraction unbound (human)	0	Fu
BBB permeability	−0.074	log BB
CNS permeability	−1.719	log PS
Metabolism		
CYP2D6 substrate	No	Yes/No
CYP3A4 substrate	Yes	Yes/No
CYP1A2 inhibitor	No	Yes/No
CYP2C19 inhibitor	No	Yes/No
CYP2C9 inhibitor	No/Yes (pkCSM/Swiss-ADME)	Yes/No
CYP2D6 inhibitor	No	Yes/No
CYP3A4 inhibitor	No	Yes/No
Excretion		
Total clearance	0.338	log mL/min/kg
Renal OCT2 substrate	No	Yes/No
Toxicity		
AMES toxicity	No	Yes/No
Max. tolerated dose (human)	0.079	log mg/kg/day
hERG I inhibitor	No	Yes/No
hERG II inhibitor	No	Yes/No
Oral rat acute toxicity (LD_50_)	2.188	mol/kg
Oral rat chronic toxicity	2.408	log mg/kg_bw/day
Hepatotoxicity	Yes	Yes/No
Druglikeness and Medicinal Chemistry		
Lipinski	Yes; 1 violation: MLOGP > 4.15	Yes/No
Ghose	No; 3 violation: WLOGP > 5.6, MR > 130, atoms > 70	Yes/No
Veber	Yes	Yes/No
Egan	No; 1 violation: WLOGP > 5.88	Yes/No
Muegge	No; 1 violation: XLOGP3 > 5	Yes/No
Bioavailability score	85	Percentage (%)
PAINS	0 alert	Number of alerts
Brenk	1 alert: isolated alkene	Number of alerts
Lead-likeness	No; 2 violations: MW > 350 XLOGP3 > 3.5	Yes/No
Synthetic accessibility	5.87	1–10 scale

## Data Availability

The data presented in this study are available in the main text of this article/supplementary materials of this article or on request from the corresponding author.

## Supplementary Materials

The following supporting information can be downloaded at https://www.mdpi.com/article/10.3390/ijms26136420/s1.

## Abbreviations

ADMET	Absorption, Distribution, Metabolism, Excretion, and Toxicity
APE1	(Apurinic or apyrimidinic site) endonuclease 1
ARE	Antioxidant responsive element
BrdU	Bromodeoxyuridine
CAII	Carbonic anhydrase 2
CADD	Computer-aided drug design
CES1	Carboxylesterase 1
CLK4	CDC like kinase 4
CPSD	Cathepsin D
DMSO	Dimethyl sulfoxide
EdU	5-Ethynyl-2′-deoxyuridine
EGFR	Epidermal growth factor receptor
FBS	Fetal bovine serum
KEAP1	Kelch-like ECH-Associating protein 1
MAPKs	Mitogen-activated protein kinases
MD	Molecular dynamics
MTT	3-(4,5-Dimethylthiazol-2-yl)-2,5-Diphenyltetrazolium Bromide
NF-κB	Nuclear factor NF-kappa-B
NRF2	Nuclear factor erythroid 2-related factor 2
OCT2	Organic Cation Transporter 2
OXA	Oxaliplatin
PARP1	Poly(ADP-ribose) polymerase-1
PBS	Phosphate-buffered saline
PLA2G2A	Phospholipase A2 Group IIA
PTGES	Prostaglandin E synthase
PTP1β	Protein tyrosine phosphatase 1β
PTPN2	T-cell protein tyrosine phosphatase
PYGL	Glycogen phosphorylase
RA	Roburic acid
RMSD	Root-mean-square deviation
RMSF	Root-mean-square fluctuation
TNF-α	Tumor necrosis factor α
TNF-R1	Tumor necrosis factor receptor 1
VDss	Volume of distribution at steady state
