# Supplementary material for: Anticancer Activity of Roburic Acid: In Vitro and In Silico Investigation
Source: Int J Mol Sci. 2025 Jul 3;26(13):6420. doi: 10.3390/ijms26136420 (PMC12250105; doi:10.3390/ijms26136420)
Supplement: Supplementary file 1 [file ijms-26-06420-s001.zip › ijms-3676341-supplementary.pdf]

Supplementary Information

Anticancer activity of roburic acid: in vitro and in silico investigation

Adrianna Gielecińska [1,2], Mateusz Kciuk [1], Somdutt Mujwar [3], Johannes A. Schmid [4], and Renata Kontek [1,*]

[1] Department of Molecular Biotechnology and Genetics, University of Lodz, Banacha 12/16, 90-237 Lodz, Poland
[2] Doctoral School of Exact and Natural Sciences, University of Lodz, Banacha Street 12/16, 90-237 Lodz, Poland
[3] Chitkara College of Pharmacy, Chitkara University, Rajpura140401, Punjab, India;
[4] Institute of Vascular Biology and Thrombosis Research, Center for Physiology and Pharmacology, Medical University of Vienna, Schwarzspanierstrasse 17, 1090, Vienna, Austria
* Correspondence: renata.kontek@biol.uni.lodz.pl

Table S1. Molecular docking results for RA with the predicted molecular targets, including binding affinities and identified interacting residues. The binding properties were compared to those of reference inhibitors co-crystallized with the target protein structure obtained from the PDB database (https://www.rcsb.org/).

**Predicted target with symbol**	**PDB id**	**Reference ligand**	**Binding affinity reported for experimental study/Key interacting resides reported from crystallographic studies**	**Binding score (affinity)**	**Interacting residues from docking validation study**	**Binding score (affinity) for RA and predicted target**	**Interacting residues**
tyrosine phosphatase 1B (PTP1β)	1C87	2-(oxalyl-amino)-4,7-dihydro-5H-thieno[2,3-*c*]pyran-3-carboxylic acid	Ki = 6.30e+4 nM Gly-22, Arg-22, Tyr-46, Val-49, Lys-120, Asp-181, Ala-217, Ile-219.	-9.2	**Tyr46, Val49,** Lys120, **Asp181, Ser216, Ala217,** Gly220, Arg221.	-6.98	**Tyr46,** Asp48, **Val49,** Lys116, **Asp181**, Phe182, **Ser216, Ala217,** Ile219.
T-cell protein tyrosine phosphatase (PTPN2)	7UAD	ABBV-CLS-484	Not available	-11.88	Tyr48, **Asp50**, Lys122, Asp182, **Phe183,** Cys216, Ser217, Ala218, Gly219, Gly221, **Gln260**, Gln264, Arg292.	-5.96	Tyr22, Arg26, **Asp50, Phe183,** Ile220, Met256, **Gln260.**
prostaglandin E synthase (PTGES)	5BQH	N-[4-(4-chlorophenyl)-1H-imidazol-2-yl]-2-(difluoromethyl)-5-{[(2-methylpropanoyl)amino]methyl}benzamide	IC_{50} = 3 nM Ile-32, Gln-36, Arg-52, His-53, Ser-127.	-7.03	**Arg70, Tyr117,** Arg126, **Ser127, Tyr130,** Thr131.	-8.52	**Arg70,** Glu77, **Tyr117, Ser127, Tyr130**.
carboxylesterase 1 (CES1)	1YA4	tamoxifen	Ki = 1.52e+4 nM Phe-101, Ser-221, Leu-304, Leu-318, Leu-363, Met-364, Leu-388, His-468.	-8.42	**Leu97,** Val254, **Leu255,** Phe303, **Leu304,** Ser305, Leu318, Ile359, **Leu363,**	-10.3	Ala93, **Leu97,** Val146, **Leu255, Leu304, Leu363, Met364,** Ile359, **His468.**

					**Met364, His468.**		
fatty acid-binding protein, adipocyte (FABP4)	6LJS	2-[(2-phenylphenyl)amino]benzoic acid	Kd = 110.6 nM Phe-16, Ser-53, Ser-55, Phe-57, Lys-58, Thr-60, Ala-75, Arg-126, Tyr-128.	-8.59	**Phe16, Met20, Ala33,** Ala36, **Pro38, Phe57, Ala75,** Ile104, Val115, Cys117, Arg126, **Tyr128.**	-9.04	**Phe16,** Tyr19, **Met20,** Val23, Val25, **Ala33, Pro38,** Met40, Ile51, **Phe57, Ala75,** Arg106, **Tyr128.**
NA-(apurinic or apyrimidinic site) endonuclease, mitochondrial (APE1)	7TC2	5-nitro-1H-indole-2-carboxylic acid	Leu-62, Ile-64, Ile-91, Ser-135, Arg-136, Gln-137, Phe-162, Ser-164, Phe-165.	-5.81	Leu62, Ser135, Arg136, **Gln137,** Ser164.	-6.26	Thr61, **Gln137,** Phe162, Phe165, Leu318.
Cathepsin D (CPSD)	4OD9	N-(3,4-dimethoxybenzyl)-N-alpha-{N-[(3,4-dimethoxyphenyl)acetyl]carbamimidoyl}-D-phenylalaninamide	IC_{50} = 58 nM Asp-33, Tyr-78, Gly-79, Phe-126, Phe-131, Ile-134, Tyr-205, Asp-231, Thr-234, Met-309, Ile-311.	-9.94	**Val31,** Asp33, **Tyr78,** Gly79, Phe126, Ala129, **Phe131,** Ile134, Tyr205, Asp234, Met309, **Ile311, Ile320.**	-7.61	**Val31, Tyr78, Phe131, Ile311, Ile320.**
dual specificity protein kinase CLK4 (CLK4)	6FYV	5-[(3-chlorophenyl)amino]benzo[c][2,6]naphthyridine-8-carboxylic acid	IC_{50} = 11 nM Leu-248, Ser-249, Ser-301, Val-326.	-9.82	Leu167, Gly168, Phe172, **Val175, Ala189, Lys191,** Val225, **Phe241, Leu244, Leu295, Val324,** Asp325.	-9.19	Glu169, **Val175, Ala189, Lys191, Phe241, Leu244, Leu295, Val324.**

glycogen phosphorylase (PYGL)	2ATI	N-(2-chloro-4-fluorobenzoyl)-N'-(5-hydroxy-2-methoxyphenyl)urea	IC_{50} = 23 nM Val-40, Lys-41, Asp-42, Asn-44 Trp-67, Tyr-75, Arg-193.	-3.6	His36, **Val40, Lys41,** Asp42, **Val45.**	-4.72	**Val40, Lys41, Val45.**
phospholipase A2 (PLA2G2A)	1KQU	6-phenyl-4(R)-(7-phenyl-heptanoylamino)-hexanoic acid	Gly-30, Gly-32, His-48, Asp-49.	-9.33	Leu2, **Phe5,** His6 Ile9, Ala17, Gly29, Val30, Gly31, Tyr51, Lys62, **Phe98.**	-11.83	**Phe5,** Cys44, His47, Asp48, Tyr51, Lys52, Ala94, **Phe98.**
carbonic anhydrase 2 (CAII)	3HS4	acetazolamide	IC_{50}: min: 0.49 nM, max: 4500 nM His-94, His-96, His-119, Val121, Phe131, Leu198, Thr199, Thr200, Pro201.	-5.91	**His94, His96, His119, Val121, Leu198,** Thr199, **Thr200, Trp209**.	-8.43	Asn62, **His94, His96, His119, Val121,** Phe131, Leu141, Val143, **Leu198, Thr200, Trp209.**
epidermal growth factor receptor (EGFR)	1M17	erlotinib	IC_{50}: min: 0.03 nM, max: 260 nM Ser-671, Gly-672, Asn-676, Leu-680, Glu-685, Tyr-740, Ser-744.	-6.74	**Leu694, Val702,** Ala719, **Lys721,** Met742, Leu764, Met769, Leu820.	-8.58	**Leu694,** Phe699, **Val702, Lys721,** Leu768, Thr830, Asp831.

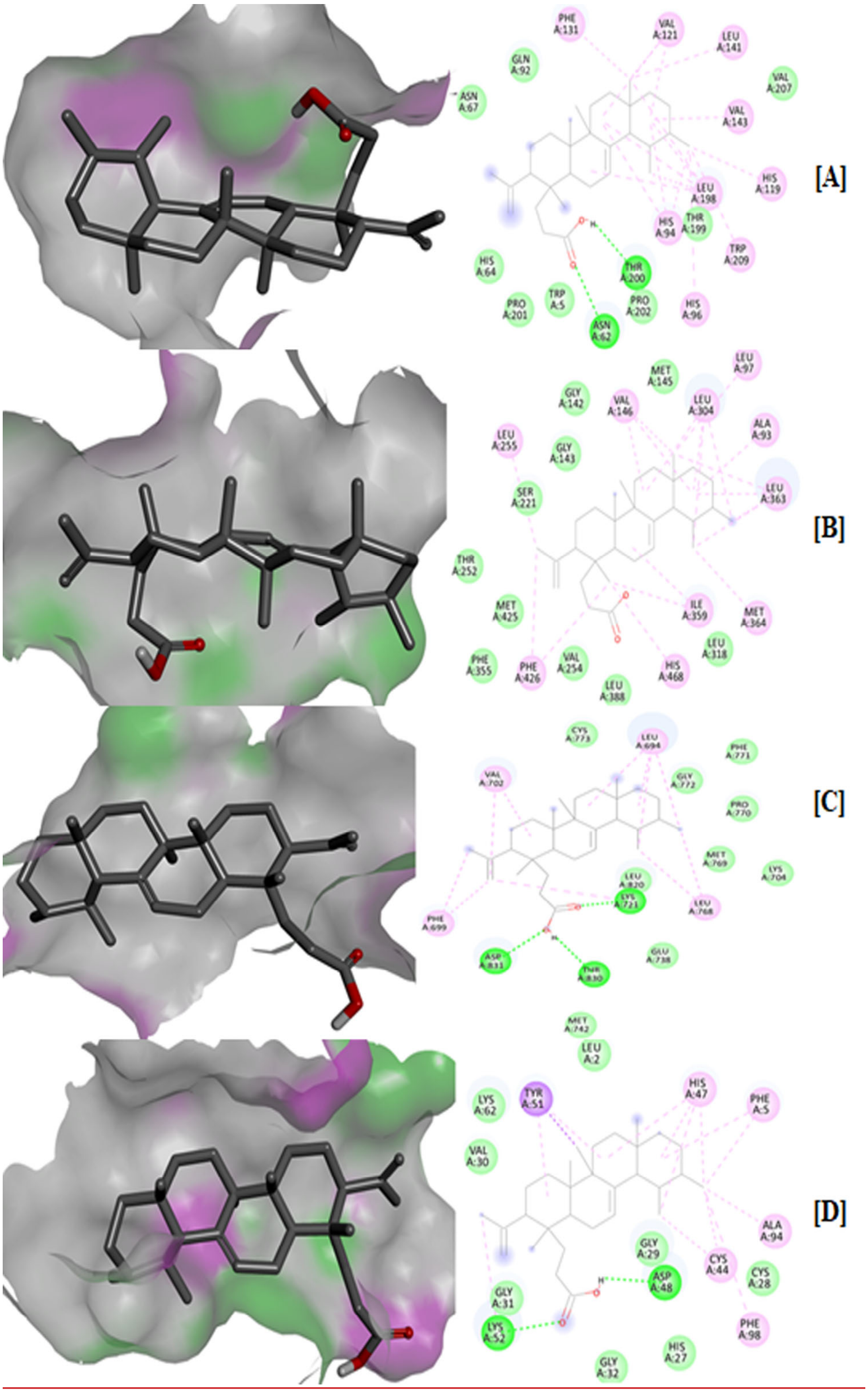

Figure S1. Two-dimensional and three-dimensional binding modes of RA with [A] CAII, [B] CES1, [C] EGFR, and [D] PLA2G2A.

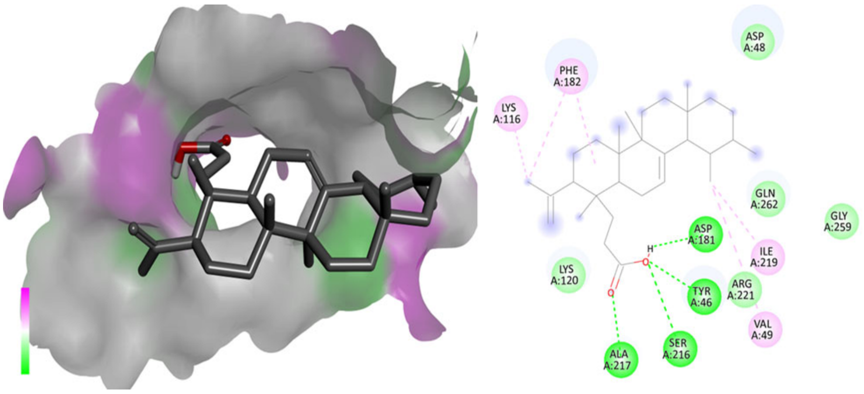

Figure S2. The two-dimensional and three-dimensional binding interactions of RA within the active site of 1c87 as predicted by molecular docking.

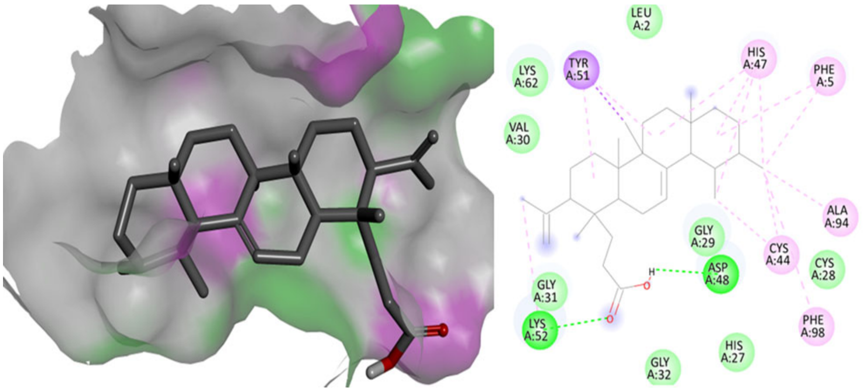

Figure S3. The two-dimensional and three-dimensional binding interactions of RA within the active site of 1kqu as predicted by molecular docking.

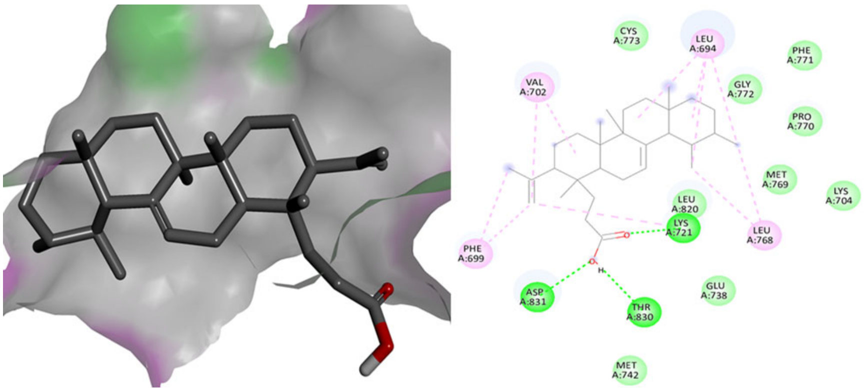

Figure S4. The two-dimensional and three-dimensional binding interactions of RA within the active site of 1m17 as predicted by molecular docking.

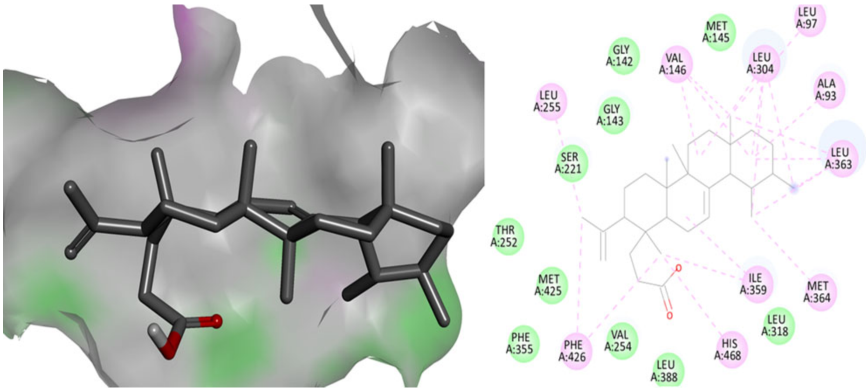

Figure S5. The two-dimensional and three-dimensional binding interactions of RA within the active site of 1ya4 as predicted by molecular docking.

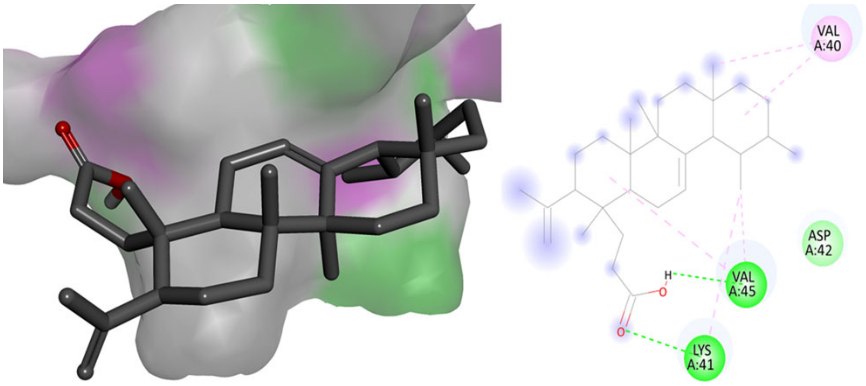

Figure S6. The two-dimensional and three-dimensional binding interactions of RA within the active site of 2ati as predicted by molecular docking.

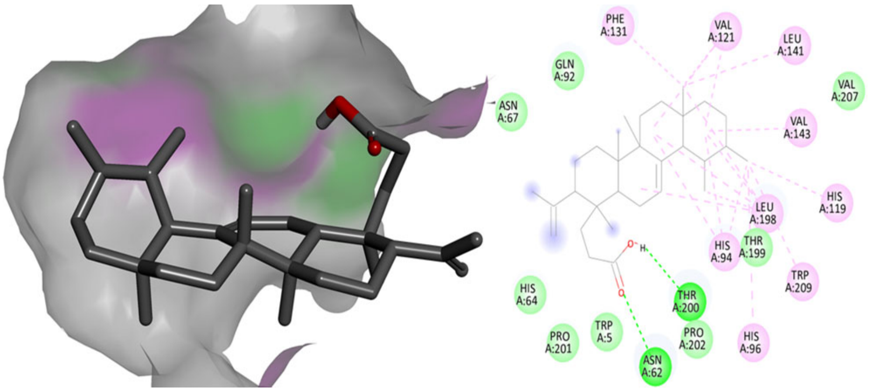

Figure S7. The two-dimensional and three-dimensional binding interactions of RA within the active site of 3hs4 as predicted by molecular docking.

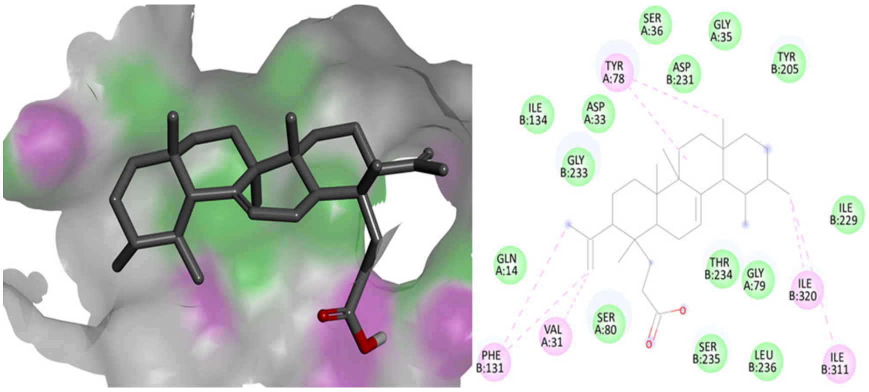

Figure S8. The two-dimensional and three-dimensional binding interactions of RA within the active site of 4od9 as predicted by molecular docking.

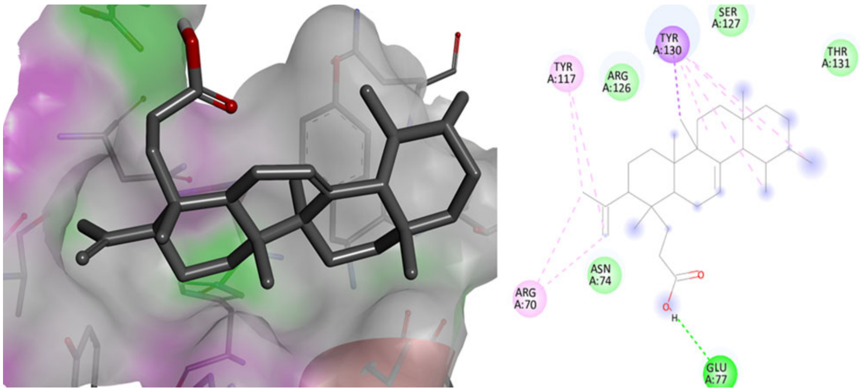

Figure S9. The two-dimensional and three-dimensional binding interactions of RA within the active site of 5bqh as predicted by molecular docking.

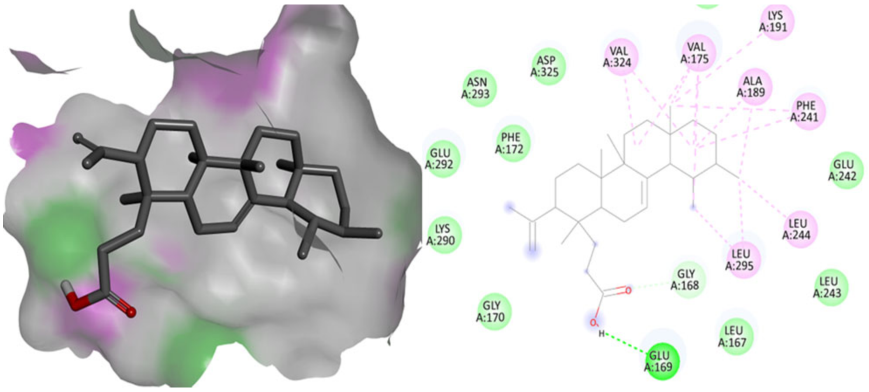

Figure S10. The two-dimensional and three-dimensional binding interactions of RA within the active site of 6fyv as predicted by molecular docking.

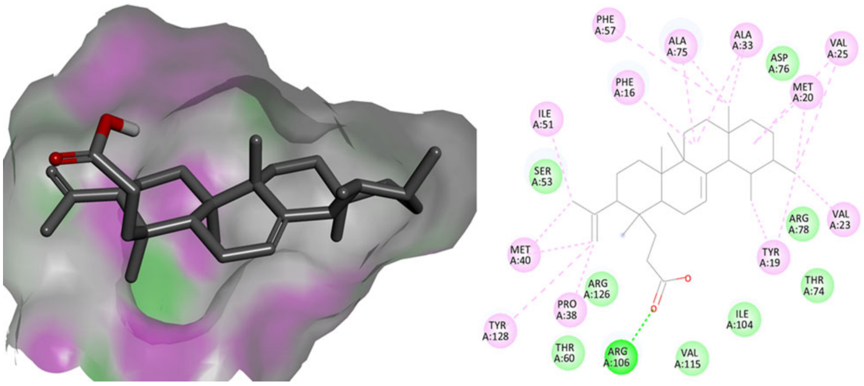

Figure S11. The two-dimensional and three-dimensional binding interactions of RA within the active site of 6ljs as predicted by molecular docking.

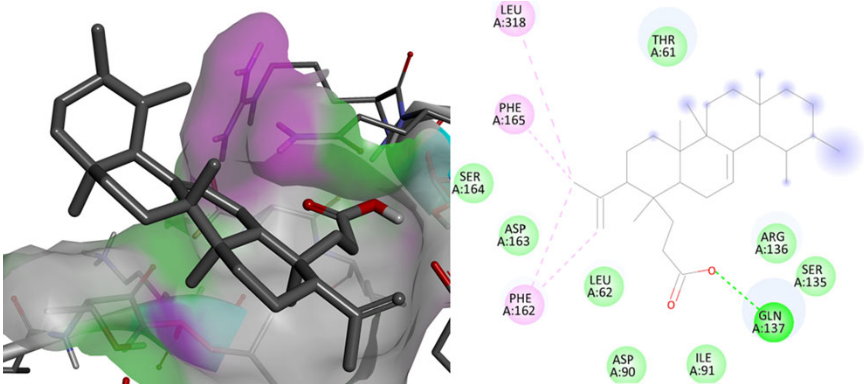

Figure S12. The two-dimensional and three-dimensional binding interactions of RA within the active site of 7tc2 as predicted by molecular docking.

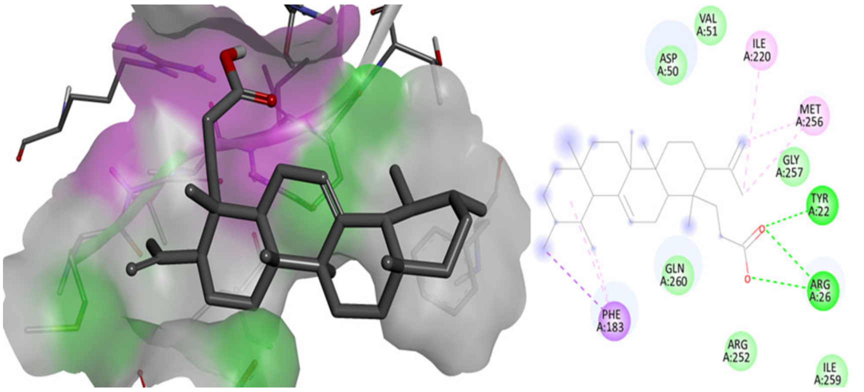

Figure S13. The two-dimensional and three-dimensional binding interactions of RA within the active site of 7uad as predicted by molecular docking.

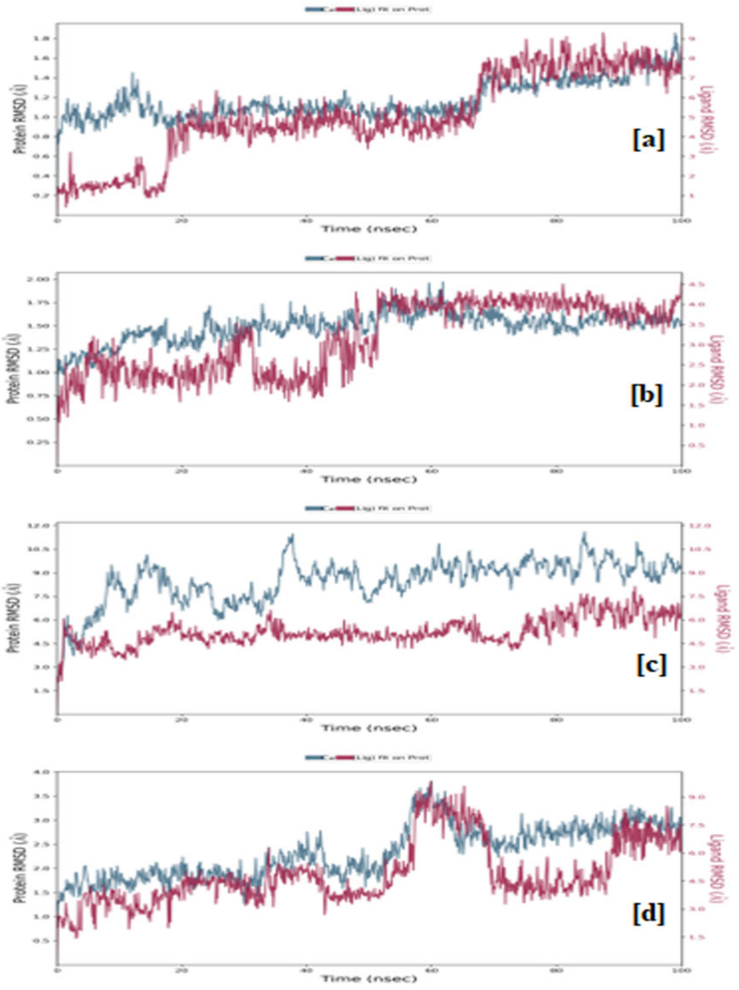

Figure S14. The RMSD for the ligand RA complexed with (a) CAII, (b) CES1, (c) EGFR, and (d) PLA2G2A detected while executing a 100 ns MD simulation.

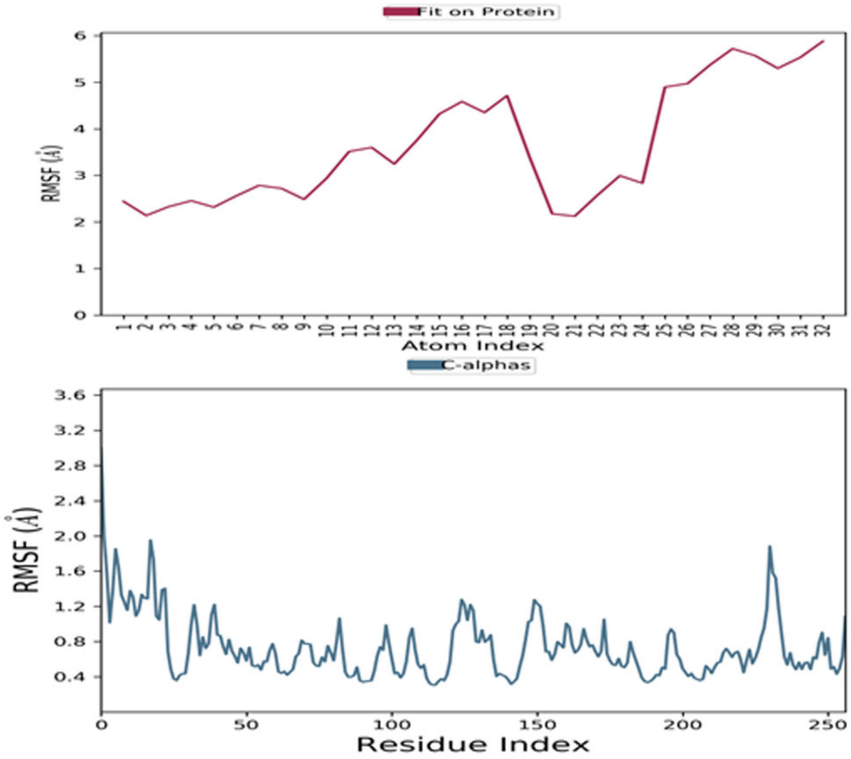

Figure S15. RMSF for the CAII complexed with ligand RA detected while executing MD simulation.

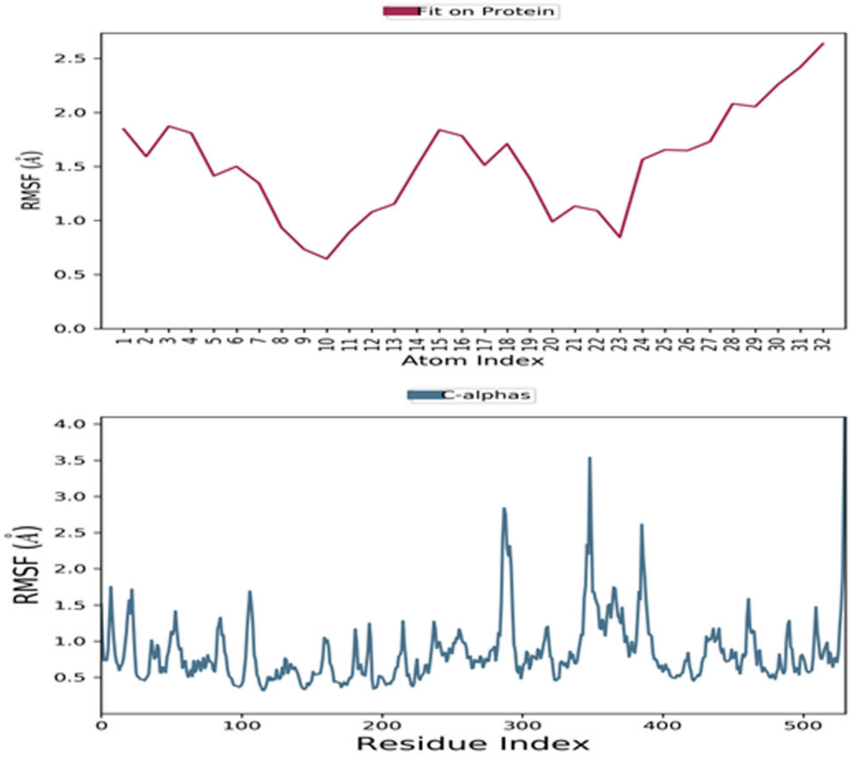

Figure S16. RMSF for the CES1 complexed with ligand RA detected while executing MD simulation.

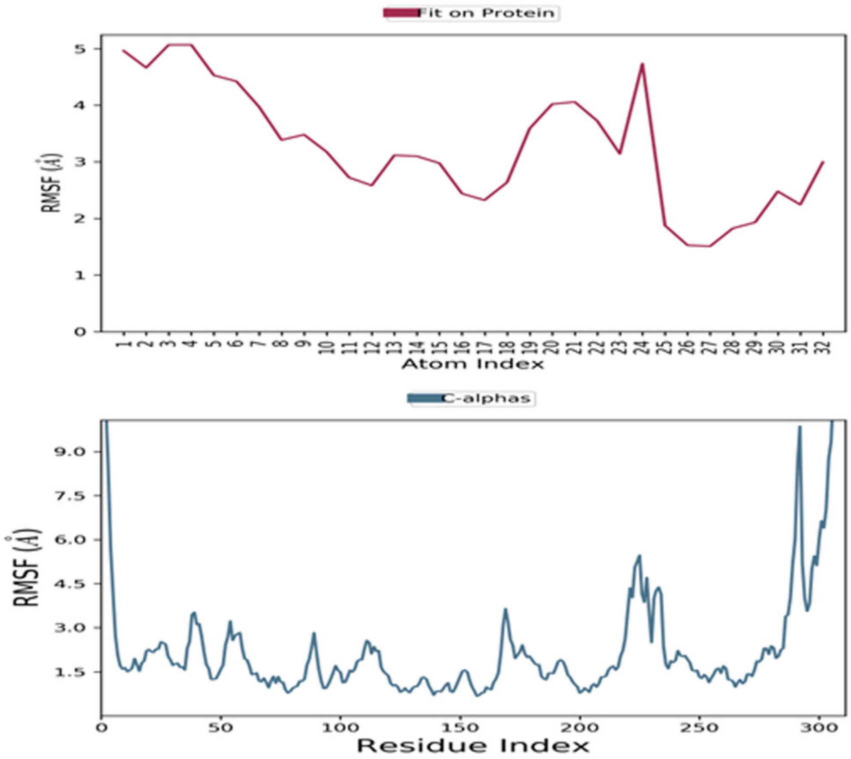

Figure S17. RMSF for the EGFR complexed with ligand RA detected while executing MD simulation.

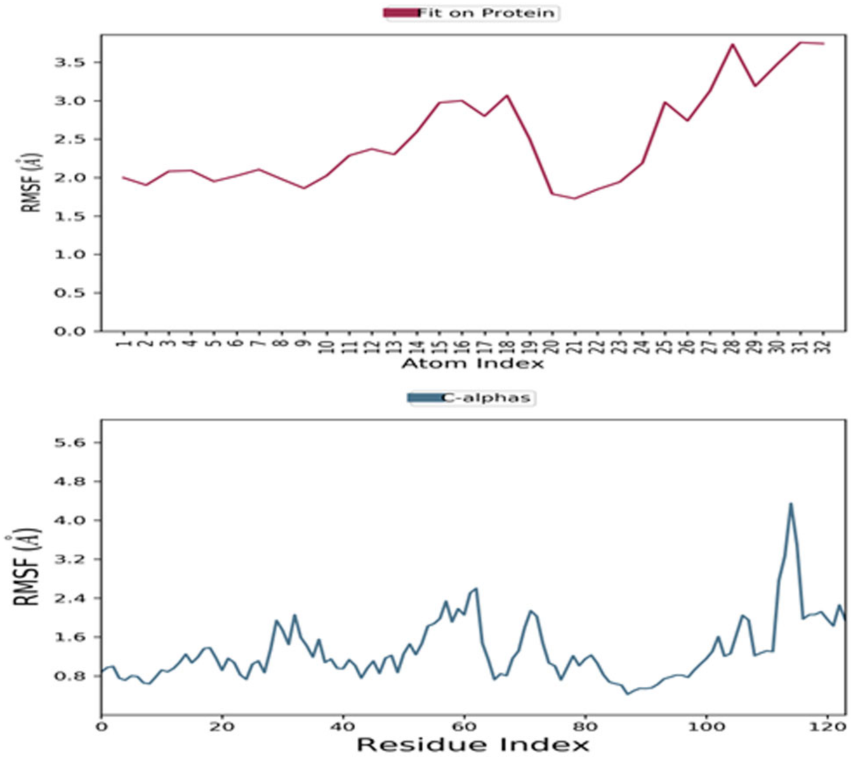

Figure S18. RMSF for the PLA2G2A complexed with ligand RA detected while executing the MD simulation.

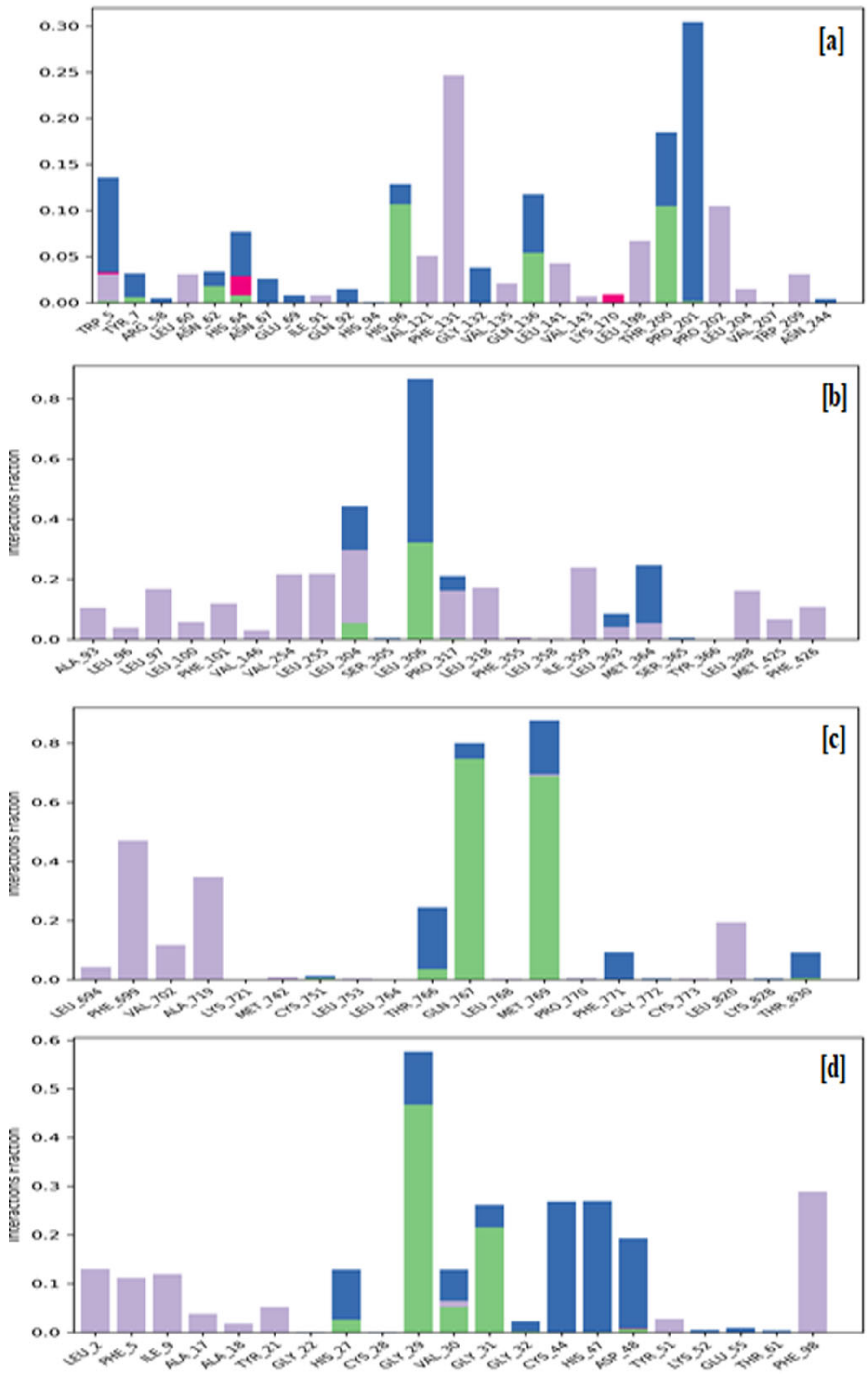

Figure S19. Protein-Ligand Contacts. The interacting residues were represented for (a) CAII, (b) CES1, (c) EGFR, and (d) PLA2G2A, receptors with ligand RA via hydrophobic interactions represented in purple-colored bars, interactions via formation of water bridges represented in blue-colored bars, and hydrogen bonds represented in green colored bars.
